# The serine/threonine kinase Stk and the phosphatase Stp regulate cell wall synthesis in *Staphylococcus aureus*

**DOI:** 10.1038/s41598-018-32109-7

**Published:** 2018-09-12

**Authors:** Marcel Jarick, Ute Bertsche, Mark Stahl, Daniel Schultz, Karen Methling, Michael Lalk, Christian Stigloher, Mirco Steger, Andreas Schlosser, Knut Ohlsen

**Affiliations:** 1 University of Würzburg, Institute for Molecular Infection Biology, Würzburg, Germany; 2 University of Tübingen, IMIT - Infection biology, Tübingen, Germany; 3 University of Hohenheim, Core Facility - Module 1 Mass Spectrometry Unit, Stuttgart, Germany; 4 University of Tübingen, Center for Plant Molecular Biology, Tübingen, Germany; 5 University of Greifswald, Institute of Biochemistry, Greifswald, Germany; 6 University of Würzburg, Imaging Core Facility - Biocenter, Würzburg, Germany; 7 University of Würzburg, Rudolf Virchow Center for Experimental Biomedicine, Würzburg, Germany

## Abstract

The cell wall synthesis pathway producing peptidoglycan is a highly coordinated and tightly regulated process. Although the major components of bacterial cell walls have been known for decades, the complex regulatory network controlling peptidoglycan synthesis and many details of the cell division machinery are not well understood. The eukaryotic-like serine/threonine kinase Stk and the cognate phosphatase Stp play an important role in cell wall biosynthesis and drug resistance in *S. aureus*. We show that *stp* deletion has a pronounced impact on cell wall synthesis. Deletion of *stp* leads to a thicker cell wall and decreases susceptibility to lysostaphin. Stationary phase Δ*stp* cells accumulate peptidoglycan precursors and incorporate higher amounts of incomplete muropeptides with non-glycine, monoglycine and monoalanine interpeptide bridges into the cell wall. In line with this cell wall phenotype, we demonstrate that the lipid II:glycine glycyltransferase FemX can be phosphorylated by the Ser/Thr kinase Stk *in vitro*. Mass spectrometric analyses identify Thr32, Thr36 and Ser415 as phosphoacceptors. The cognate phosphatase Stp dephosphorylates these phosphorylation sites. Moreover, Stk interacts with FemA and FemB, but is unable to phosphorylate them. Our data indicate that Stk and Stp modulate cell wall synthesis and cell division at several levels.

## Introduction

*Staphylococcus aureus* is an opportunistic pathogen that inhabits the human skin and mucosa and causes a large variety of nosocomial and community-acquired infections^1^. Nowadays, it is difficult to treat *S. aureus* infections because *S. aureus* has acquired resistance to multiple drugs, including penicillin, methicillin and vancomycin^2^. Therefore, there is a need for new antimicrobial drugs against *S. aureus* and its multiple antibiotic-resistant strains. The most promising strategy to combat antibiotic resistance is to find novel antibiotics which interfere with the cell wall biosynthesis pathway^3^.

**Figure 1:**
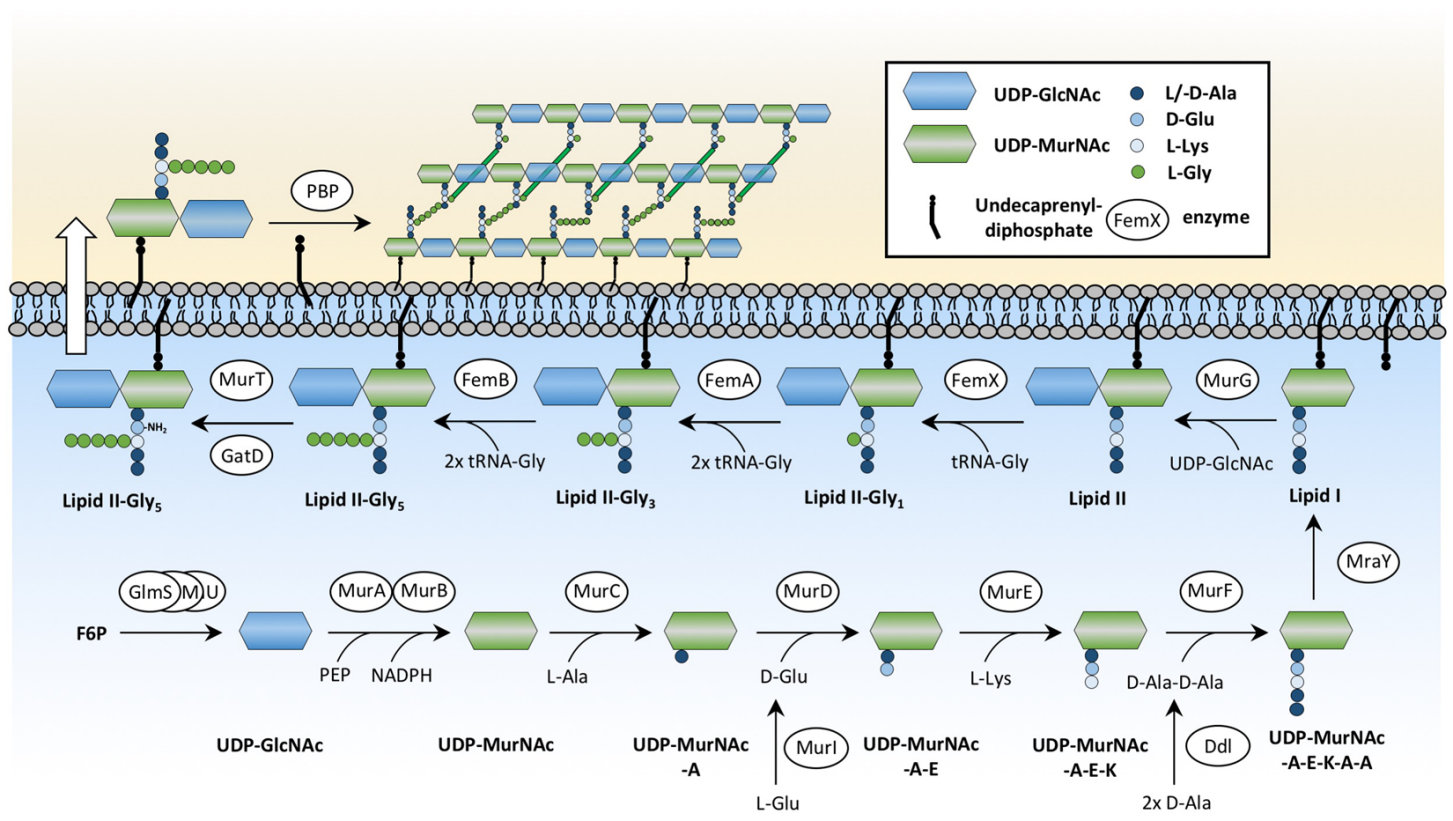
Peptidoglycan synthesis pathway of *S. aureus*. The PGN synthesis of *S. aureus* starts with glucosamine-6-phosphate (GlcN6P) as the central metabolite controlling cell wall synthesis and glycolysis. The aminotransferase GlmS converts fructose-6- phosphate (F6P) into GlcN6P using glutamine as a nitrogen source. GlcN6P is processed to *N*-acetylglucosamine (UDP-GlcNAc) by the activity of the phosphoglucosamine mutase GlmM and the uridyltransferase GlmU^60^. The conversion of UDP-GlcNAc to *N*-acetylmuramic acid (UDP-MurNAc) is catalyzed by MurA and MurB. Mur ligases (MurC-F) sequentially add the amino acids L-Ala, D-Glu, and L-Lys and the dipeptide D-Ala-D-Ala to UDP-MurNAc, yielding Park’s nucleotide (UDP-MurNAc-L-Ala-D-Glu-L-Lys-D-Ala-D-Ala). This soluble PGN precursor is then linked to the lipid carrier undecaprenyl-diphosphate via the membrane translocase MraY, producing membrane-bound lipid I. The membrane glycosyltransferase MurG links UDP-GlcNAc to the lipid I molecule, yielding lipid II (undecaprenyl-diphosphate-GlcNAc-MurNAc-pentapeptide). In *S. aureus*, lipid II is further modified by the addition of five glycine residues catalyzed by the FemX/A/B proteins. These non-ribosomal peptidyl-transferases use glycyl-tRNAs to sequentially add five glycine’s to the PGN-lysyl side chain of lipid II^61^. FemX^62,63^ adds the first glycyl unit, FemA^63,64^ the second and third unit, and FemB^40,63^ adds the fourth and fifth glycyl unit to complete the pentaglycine-bridge. After specific modifications such as deamination of D-Glu of the stem peptide by MurT/GatD^65,66^, the pentaglycine-lipid II unit is translocated from the inner side to the outer side of the membrane. As the final stage of PGN synthesis, penicillin binding proteins (Pbp) incorporate extracellular lipid II into the growing PGN sacculus through transglycosylation and transpeptidation.

The bacterial cell envelope is essential for survival and pathogenicity. It forms a barrier against environmental stresses and contributes to virulence and antibiotic resistance. The cell wall of gram-positive bacteria is composed of a multi-layered mesh of cross-linked peptidoglycan (PGN). PGN consists of chains of repeating disaccharide units comprising *N*-acetylglucosamine (GlcNAc) and *N*-acetylmuramic acid (MurNAc). The lactoyl group of MurNAc is supplemented with a penta stem peptide (L-Ala-D-isoGlu-L-Lys-D-Ala-D-Ala). The staphylococcal PGN polysaccharide chains are highly cross-linked via interpeptide bridges of five glycyl residues protruding from the L-lysine of the stem-peptides^4^. These interpeptide bridges are synthesized by the FemX/A/B enzymes^5^. The overall PGN synthesis can be divided into three distinct stages: (1) formation of the nucleotide sugar-linked UDP-MurNAc-pentapeptide (Park’s Nucleotide), (2) transfer of the soluble PGN precursor to the lipid carrier undecaprenyl-diphosphate (resulting lipid I) and addition of UDP-GlcNAc, finally yielding lipid II, and (3) transport of this complete subunit across the cytoplasmic membrane and incorporation into the growing PGN sacculus mainly by penicillin-binding proteins^6^. At this final stage, cross-bridge formation occurs together with secondary modification of the newly synthesized PGN. An overview of the PGN synthesis pathway is shown in Fig. 1.

The cell wall synthesis pathway producing PGN is highly coordinated and tightly regulated^7^. There are several reports showing that next to two-component systems^8,9^, serine/threonine phosphorylation plays an important role in cell wall metabolism^10,11^. In *S. aureus* the conserved eukaryotic-like serine/threonine kinase Stk (alternatively named as PknB or Stk1) and the cognate phosphatase Stp impact bacterial cell signalling, central metabolism^12–14^, stress response^15,16^, antibiotic resistance^16–18^ and virulence^16,17,19–21^. Recently, pentaglycine-lipid II has been found to serve as a signal for activation of serine/threonine kinase Stk of *S. aureus*^9^. Pentaglycine-lipid II interacts with the extracellular PASTA (penicillin binding protein and serine/threonine kinase associated) domains of Stk and triggers its kinase activity *in vitro*^9^. In fact, deletion of *stp* and *stk* in *S. aureus* causes cell division defects resulting in the formation of multiple and incomplete septa, differences in cell size and cell wall thickness^10,22^. In addition, *stk* and *stk/stp* deletion strains are more susceptible to cell wall-acting antibiotics like tunicamycin^12^, fosfomycin^12,20^ and β-lactam antibiotics^10,16^. Moreover, the phosphatase Stp contributes to reduced susceptibility to vancomycin and enhanced virulence^23^. Furthermore, Stk cross-talks with two-component systems involved in cell wall metabolism by phosphorylation of the response regulator of VraTSR^8^, WalRK^9^ and GraSR^24^, affecting the expression of the cell wall stimulon and cell wall hydrolases as well as the cell wall charge. There are also studies which have shown that Stk homologs regulate cell wall synthesis and cell division in *Streptococcus*^25–28^, *Bacillus*^29,30^, *Enterococcus*^31^ and *Streptomyces*^32^.

However, there is still a lack of knowledge about the Stk- and Stp-dependent regulation in cell physiology. In this study, we specify the role of Stk and Stp in regulating cell wall synthesis by analysis of the cell wall phenotype of *stk*, *stp*, and *stk/stp* mutant strains. Deletion of *stp* leads to a thicker cell wall with incomplete muropeptides and reduced susceptibility to lysostaphin. In addition, we discover that the essential cell wall synthesis enzyme FemX is a target of Stk and Stp. Moreover, we show that Stk interacts with FemA/B and other cell wall synthesis and cell division proteins.

## Results

### *stp* deletion leads to an altered muropeptide composition in the stationary phase

**Figure 2:**
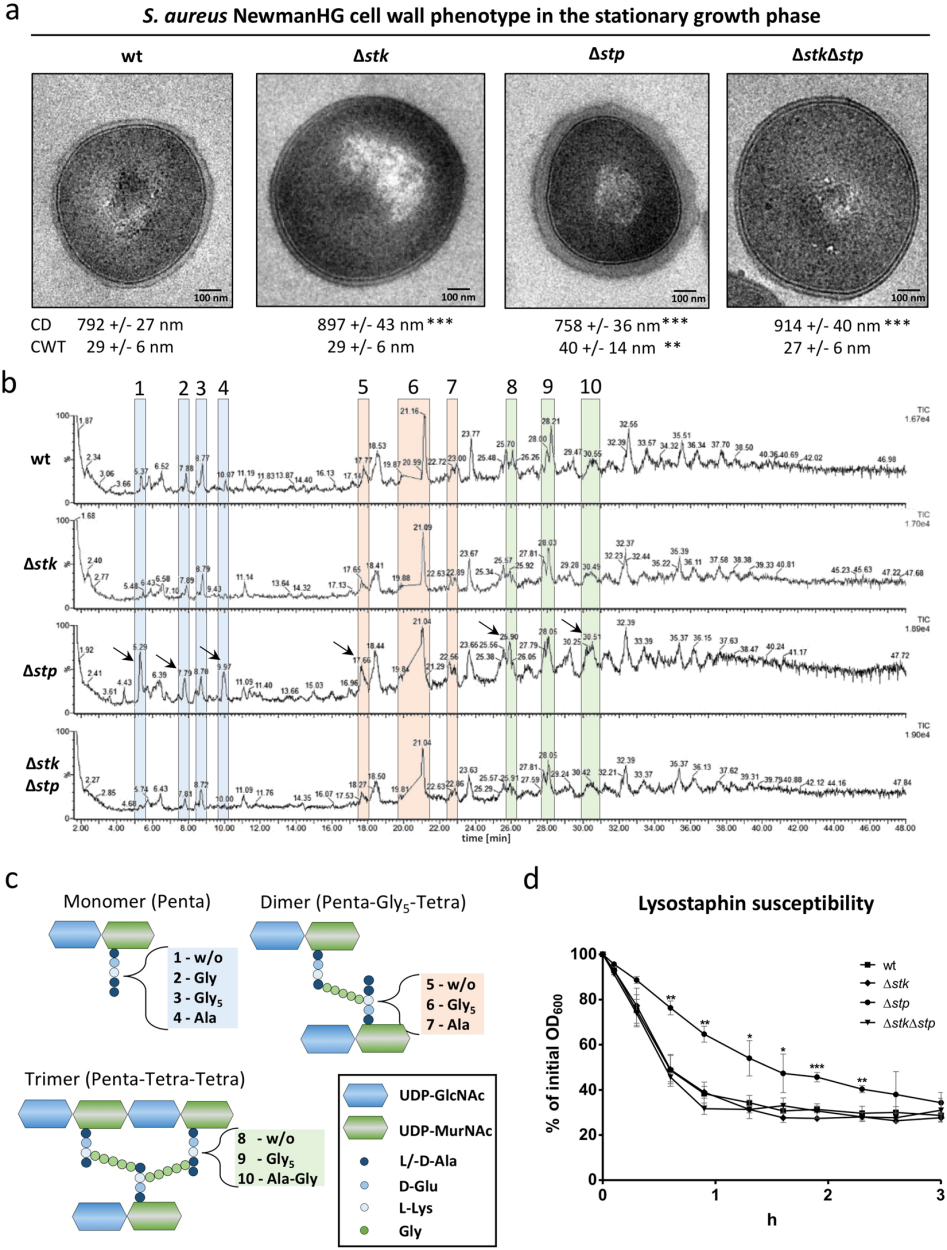
Cell wall phenotype of *S. aureus* NewmanHG wt, Δ*stk*, Δ*stp* and Δ*stk*Δ*stp* strains at stationary growth phase. (**a**) Analysis of cell morphology and cell wall thickness of *S. aureus* wt and mutant cells at the same stage in the cell cycle by TEM. The mean cell diameter (CD) and the cell wall thickness (CWT) was assessed based on the diameter of the 20 largest cells out of 100 cells per strain. The CWT was measured at five different points on the cell wall. (**b**) Muropeptide profile of *S. aureus* wt and mutant strains obtained by UPLC-MS. Highlighted muropeptide peaks were identified by MS and their structures are shown in (**c**). The chromatogram of *S. aureus* Δ*stp* at stationary phase showed an accumulation of monomers (peak 1, 2, 4), dimers (peak 5, 7) and trimers (peak 8, 10) with incomplete interpeptide bridges. (**d**) Lysostaphin susceptibility of *S. aureus* wt and mutant strains. Data represent percentages of the initial optical density (OD_600_) over three hours after treatment with 0.5 µg/ml lysostaphin. The graphs show the mean and standard deviation of three independent experiments. Significance values of CD, CWT and lysostaphin susceptibility were calculated using a two-tailed unpaired student t-test (*p < 0.05, **p < 0.01, ***p < 0.001).

To determine the role of Stk and Stp in cell wall metabolism we analysed morphological differences and the cell wall composition of *S. aureus* NewmanHG wild type and *stk*, *stp* and *stk/stp* deletion strains (Δ*stk*, Δ*stp*, and Δ*stk*Δ*stp*) in logarithmic and stationary phase cultures. First, we investigated the cell wall morphology of the deletion strains in *S. aureus* NewmanHG background by TEM, since previous reports have demonstrated severe cell wall structural alteration in *S. aureus* strains N315^10^ and MW2^22^. In the stationary phase, *stk* and *stk*/*stp* mutant cells were up to 15% larger in diameter than wild type cells. In contrast, *stp* mutant cells were 4% smaller (Fig. 2a) in the stationary phase than wild type cells. Logarithmic phase cells were generally larger (10%) than stationary phase cells. In the logarithmic phase, Δ*stk*, Δ*stp* and Δ*stk*Δ*stp* were significantly larger than wild type cells (8%, 7% and 16%, respectively) (Fig. S1a). The cell walls of stationary phase *stp* mutant cells were significantly thicker (38%) compared to the other strains (Fig. 2a). In logarithmic phase, the cell wall of Δ*stk* was significantly thinner (23%), whereas the cell wall of Δ*stp* was thicker (26%) than the one of the wild type strain or double mutant (Fig. S1a). Moreover, we observed morphological alterations like detached cell wall or membrane-like fragments in Δ*stk* and Δ*stk*Δ*stp* cells particularly at logarithmic phase. A similar observation was reported previously for stationary phase cells in another strain background^10^. The most prominent result to emerge from these electron microscopy data is the thicker cell wall of the *stp* deletion strain.

To identify molecular patterns associated with the observed changes in cell wall thickness, we isolated PGN of all four strains and digested it with the muramidase mutanolysin, which hydrolyses the sugar chains into disaccharides. The resulting muropeptides were analysed by UPLC as well as by UPLC-MS. This enabled us to directly determine the masses and the potential structures of the muropeptide peaks (Fig. S2a). The muropeptide chromatograms of all strains at both growth phases were virtually the same (Fig. S1b), with only one exception: There was a strong increase of several muropeptide peaks of PGN isolated from the *stp* mutant in stationary phase (Fig. 2b,c). UPLC-MS analysis proved that this strain still synthesized classical muropeptides: the monomer Penta-Gly_5_ (peak 3), the cross-linked dimer Penta-Gly_5_-Tetra-Gly_5_ (peak 6) and trimer Penta-Gly_5_-Tetra-Gly_5_-Tetra-Gly_5_ (peak 9). However, we found additional muropeptides with changes within the interpeptide bridge in stationary Δ*stp* cells. Either the pentaglycine was exchanged by a single glycine (peak 2: Penta-Gly), by alanine (peak 4: Penta-Ala and peak 7: Penta-Gly_5_-Tetra-Ala) or by Ala-Gly (peak 10: Penta-Gly_5_-Tetra-Gly_5_-Tetra-Ala-Gly). Moreover, one pentaglycine bridge was missing completely, as in peak 1 (Penta), peak 5 (Penta-Gly_5_-Tetra) and peak 8 (Penta-Gly_5_-Tetra-Gly_5_-Tetra). When we searched for the respective masses of these incomplete muropeptides in the other strains, they were much less abundant or even non-detectable (Fig. S2b). In contrast, the amount of the classical muropeptides was within the same range in all four strains tested in both growth phases. Importantly, incomplete muropeptides were accumulated exclusively in stationary *stp* mutant cells compared to all three other strains (Fig. S2b). This shows that the incomplete muropeptides are caused by the *stp* deletion. The PGN of *S. aureus* is highly cross-linked. The amount of PGN cross-linking was not significantly different in any of the four strains showed no significant differences (Fig. S2c) and was within the typical range (73%)^5^. There were no differences in the muropeptide composition of the *stp* strain regarding deacetylation of the sugar moieties or amidation of glutamate compared to the wild type strains.

### *S. aureus* Δ*stp* is less susceptible to lysostaphin

Based on the altered cell wall thickness and cell wall composition of the Δ*stp* strain, we suspected that deletion of *stp* directly affected the stability of the cell wall. Therefore we determined the susceptibility of each strain to the glycyl-glycine endopeptidase lysostaphin by measuring the decline in optical cell density. The Δ*stp* strain was less susceptible to lysostaphin compared to the other strains at both growth conditions (Fig. 2d). After lysostaphin treatment of stationary phase cells, the Δ*stp* strain reached 50% lysis later (85 min) compared to wt, Δ*stk* and Δ*stk*Δ*stp* strain (35 min). During the logarithmic phase (Fig. S1d), Δ*stp* cells needed 78 min, Δ*stk* needed 60 min, wild type and Δ*stk*Δ*stp* needed 35 min to reach 50% lysis due to lysostaphin treatment. Interestingly, in the logarithmic phase the Δ*stp* strain was more susceptible to lysostaphin (78 min) compared to stationary phase Δ*stp* (85 min). The most striking result to emerge from the data is the reduced lysostaphin susceptibility of Δ*stp* mutant in both growth phases.

### *stp* deletion leads to accumulation of cell wall metabolites

**Figure 3:**
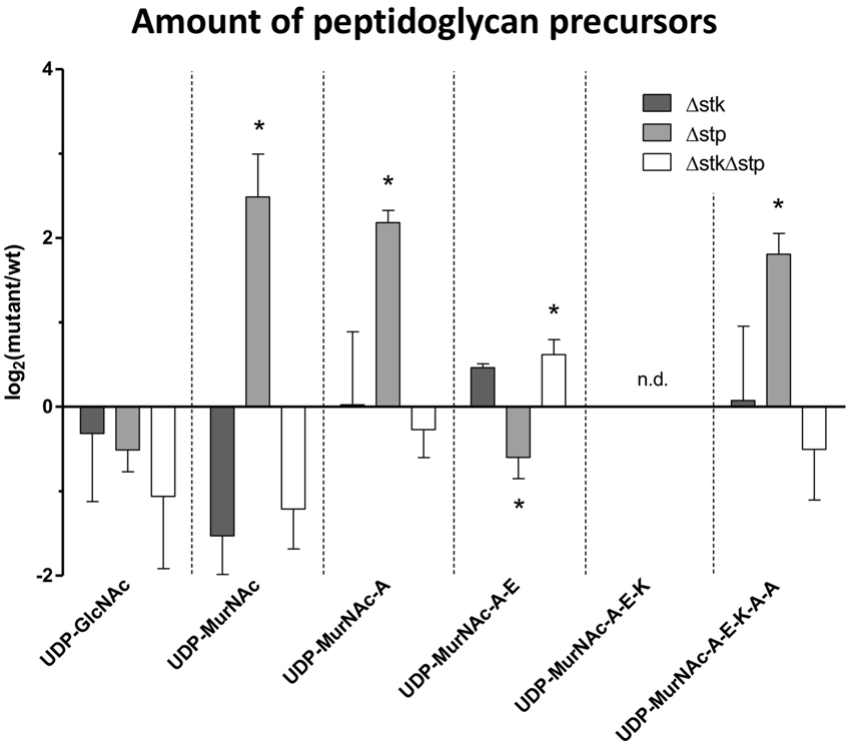
Stk and Stp affect intracellular peptidoglycan precursor concentration. Bar charts represents the log_2_-fold changes of respective metabolites from Δ*stk*, Δ*stp* and Δ*stk*Δ*stp* mutants compared to wild type cells at stationary phase. Significant differences (non-parametric unpaired Mann-Whitney test; *p < 0.05) are marked with an asterisk. A alanine, E glutamic acid, K lysine, n.d. not detectable.

In order to explain the muropeptide diversity of the thick cell wall phenotype of Δ*stp*, the cell wall metabolites were investigated in all strains using LC-MS. All soluble cell wall precursors, beginning with UDP-GlcNAc and UDP-MurNAc and ending with Park’s Nucleotide (UDP-MurNAc-Ala-Glu-Lys-Ala-Ala), were detected in all strains in both growth conditions. The significant differences between wild type and Δ*stk* or Δ*stp* mutant strains in logarithmic and stationary phase cells are discussed below (Figs 3 and S3). As expected, the abundance of cell wall metabolites was higher in the logarithmic phase- than in stationary phase cells (Fig. S3). In logarithmic phase cells, UDP-GlcNAc (2.336 to 3.103) was the most abundant cell wall precursor in all strains. In contrast, UDP-MurNAc-Ala-Glu-Lys (0.003 to 0.008) was less abundant in all strains in the logarithmic phase (Fig. S3a). In stationary phase cells, all cell wall precursors, with the exception of UDP-MurNAc (0.048 to 0.799), were less abundant (Fig. S3b). UDP-MurNAc-Ala-Glu-Lys was below the detection limit in stationary phase cells.

As shown in Fig. S3c, Δ*stk* and Δ*stk*Δ*stp* strains in the logarithmic phase showed a significant accumulation of UDP-MurNAc-Ala (6.0- and 6.5-fold, respectively) and UDP-MurNAc-Ala-Glu (2.6- and 2.3-fold, respectively) in comparison to wild type cells. The cell wall precursors UDP-MurNAc-Ala-Glu-Lys and UDP-MurNAc-Ala-Glu-Lys-Ala-Ala of logarithmic Δ*stk* and Δ*stk*Δ*stp* cells showed no significant changes compared to wild type cells. In contrast, in the *stp* mutant UDP-MurNAc-Ala (2.3-fold), UDP-MurNAc-Ala-Glu-Lys (2.0-fold) and UDP-MurNAc-Ala-Glu-Lys-Ala-Ala (2.6-fold) were accumulated significantly in comparison to the wild type. In the stationary phase (Figs 3 and S3d), Δ*stk* and Δ*stk*Δ*stp* strains showed no significant changes in cell wall precursor abundance in comparison to wild type cells, with the exception of UDP-MurNAc-Ala-Glu (1.5-fold increase) in Δ*stk*Δ*stp* cells. Interestingly, the Δ*stp* strain showed a significant accumulation of UDP-MurNAc (5.5-fold), UDP-MurNAc-Ala (4.6-fold) and UDP-MurNAc-Ala-Glu-Lys-Ala-Ala (3.5-fold). In contrast, UDP-MurNAc-Ala-Glu was slightly decreased (1.5-fold) in stationary Δ*stp* cells. The most distinct aspect of the metabolomics data is that *stp* mutant cells accumulate the cell wall precursors UDP-MurNAc-Ala to UDP-MurNAc-Ala-Glu-Lys-Ala-Ala in the logarithmic phase and UDP-MurNAc, UDP-MurNAc-Ala and UDP-MurNAc-Ala-Glu Lys-Ala-Ala in the stationary phase. On the other hand, in Δ*stk* and Δ*stk*Δ*stp* mutants UDP-MurNAc-Ala and UDP-MurNAc-Ala-Glu accumulate in the logarithmic growth phase, whereas the early PGN precursors UDP-GlcNAc and UDP-MurNAc decrease in the stationary growth phase.

### Stk interacts with cell wall synthesis- and cell division proteins

**Figure 4:**
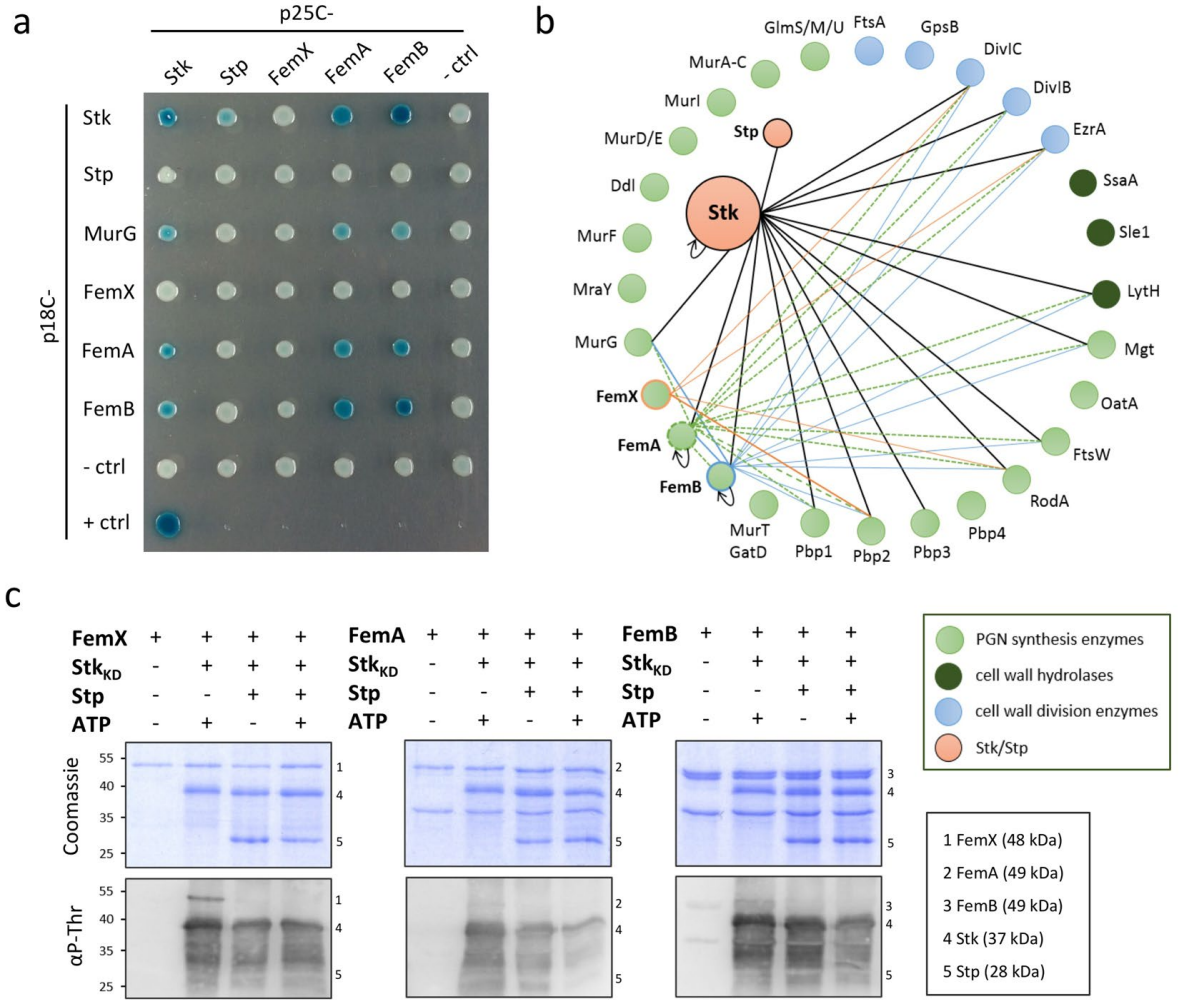
Interaction network of Stk and Stp as well as FemX/A/B. (**a**) Stk as well as FemA and FemB formed homodimers and interacted with each other and as well as with MurG. For bacterial two-hybrid analysis, co-transformants of *cya* mutant *E. coli* BTH101 were spotted onto X-gal indicator plates. The blue colour after 5 days incubation time at 18 °C indicated the reconstitution of the functional adenylate cyclase due to the interaction of the fusion proteins. pKT25-*zip*/pUT18-*zip* represented the positive control, whereas empty plasmids p25C and p18C represented negative controls. Every interaction was tested in at least three independent experiments. (**b**) Interaction network of Stk, Stp and FemX/A/B proteins among cell wall synthesis and cell division proteins as determined by bacterial two-hybrid analysis (Fig. S4). Interaction is indicated by a line. Homodimerization of Stk, FemA and FemB is indicated by a circular arrow. (**c**) Stk phosphorylated FemX but did not phosphorylate FemA or FemB in the *in vitro* kinase assay. Stp dephosphorylated Stk-dependent phosphopeptides of FemX. The upper panel shows a loading control stained with Coomassie-blue; the bottom panel shows a Western blot of phosphorylated proteins using an anti-phosphothreonine antibody (αP-Thr).

Based on the changes in cell wall precursor abundance in the mutant strains and the uniquely composed cell wall of Δ*stp*, we speculated that Stk and Stp regulate cell wall synthesis enzymes. Thus, we analysed protein-protein interaction of Stk or Stp with cell wall synthesis- and cell division proteins using a bacterial two-hybrid system. All protein-protein interactions are shown in Figs 4a, S4 and S13 and are summarized in Fig. 4b. The membrane-located kinase Stk dimerized and interacted weakly with the cognate phosphatase Stp (Fig. 4a). After 5 days incubation on X-gal indicator plates, the weak Stk-Stp interaction was seen in two-thirds of the experiments. Stk interacted with membrane-associated cell wall synthesis enzymes (MurG, FemA, FemB, Pbp1, Pbp2 and Pbp3), the cell wall hydrolase LytH, the monofunctional transglycosylase Mgt, glycosyltransferases (RodA, FtsW) and cell division proteins (DivIB, DivIC, EzrA). In this approach, Stk interacted neither with cytosolic cell wall synthesis enzymes (GlmS, GlmM, GlmU, MurA-F, MurI, Ddl and FemX) nor with Pbp4, cell wall hydrolases Sle1 and SsaA, the acetyltransferase OatA nor cell division-related proteins GpsB and FtsW. The phosphatase Stp did not interact with cell wall synthesis- and cell division proteins under the tested conditions. We focused on the interaction of Stk/Stp with FemX/A/B enzymes, as the latter produce the pentaglycine-interpeptide bridge of the lipid II molecule. FemX interacted neither with Stk, Stp, FemA nor FemB. However, FemX interacted weakly with Pbp2, RodA, DivIC and EzrA. FemA and FemB interacted with Stk and with cell wall synthesis enzymes (MurG, Pbp1, Pbp2), Mgt, LytH, RodA, FtsW and cell division proteins (DivIB, DivIC, EzrA). FemA and FemB interacted with each other and also formed homodimers, which was not the case for FemX. Overall, these results indicate that the membrane-associated kinase Stk interacts with several cell wall synthesis- and cell division proteins.

### Stk and Stp phosphorylate and dephosphorylate FemX

Based on the Stk interaction with FemA/B, we speculated that Stk phosphorylates and Stp dephosphorylates the FemX/A/B proteins. Therefore, we performed *in vitro* kinase- and phosphatase assays with N-terminal polyhistidine-tagged fusion proteins (Fig. 4c). Due to the strong degradation of full-length Stk, we used the Stk kinase domain (Stk_KD_) lacking the transmembrane and extracellular PASTA domains^33^. Stk_KD_ phosphorylated FemX but not FemA or FemB. Mass spectrometry analysis revealed the Stk-mediated phosphorylation sites on FemX (Fig. S5a–e) as Thr32, Thr36 and Ser415 (Fig. S5e, peak 4). We detected mono- (Fig. S5e, peak 1 and 2) and diphosphorylated (Fig. S5e, peak 3) phosphothreonine peptides of FemX. Stp was able to completely dephosphorylate these Stk-mediated phosphorylation sites (Figs 4c and S5c). The negative control FemX alone, as well as Stk-FemX without ATP, showed only isolated phosphopeptides which were neglectable (Figs 4c and S5a). Western blot and mass spectrometry analysis showed that Stk did not phosphorylate FemA and FemB (Figs 4c and S5f–k). These data suggest that Stk phosphorylates FemX but not FemA or FemB. Stp dephosphorylates the Stk-mediated phosphorylation sites of FemX.

### *S. aureus* Δ*stk* and Δ*stk*Δ*stp* are more susceptible to cell wall-acting antibiotics

In order to localize the potential targets of Stk and Stp in the cell wall biosynthesis pathway, we determined the minimal inhibitory concentration (MIC) of cell wall-active antibiotics. We tested the susceptibilities of *S. aureus* wild type and its isogenic mutant strains to ten antibiotics which interfere with different steps of the cell wall synthesis pathway (Table S6). The MIC of the inhibitors of the intracellular cell wall precursor synthesis pathway (fosfomycin, D-cycloserine and tunicamycin) observed for the Δ*stp* strain were 2-fold lower as compared to the wild type strain. On the other hand, the MIC for the lantibiotic nisin of Δ*stp* was 2-fold higher than the wild type. The other cell wall-active antibiotics that we tested did not affect the MIC of the Δ*stp* strain. The MIC of the MurA-inhibitor fosfomycin showed a 2-fold increase for the Δ*stk* strain and a 2-fold decrease for the Δ*stk*Δ*stp* strain compared to the wild type. Interestingly, tunicamycin, an inhibitor of MraY translocase, was 8-fold more active against Δ*stk* (as already described by Donat *et al*.^12^ in the strain 8325 background) and Δ*stk*Δ*stp* strains than against the wild type. The Δ*stk* und Δ*stk*Δ*stp* strains displayed a 2-fold increased susceptibility to ramoplanin, methicillin and penicillin G compared to the wild type strain. The mutation of *stk* or *stp* did not affect the MIC of the other tested cell wall-active antibiotics such as vancomycin, daptomycin and bacitracin (Table S6).

## Discussion

Proper cell wall synthesis is essential to protect bacteria against the extracellular environment and for resisting high intracellular turgor. Synthesis of the cell wall is a complex and highly coordinated process both temporally and spatially. Although the major components of bacterial cell walls have been known for decades, many details of the complex regulatory network controlling PGN synthesis and the cell division machinery are not well understood and are a topic of current research^7^. In particular, the regulatory impact and interplay of two-component systems such as VraRS, GraSR, WalKR and the role of the eukaryotic-like serine/threonine kinase Stk and its associated phosphatase Stp remains to be discovered. Several former studies suggest an important role of Stk in cell wall metabolism^11^, cell division and autolysis^10^.

In this report we provide evidence that both the kinase Stk and the phosphatase Stp are involved in the regulation of cell wall synthesis. Notably, *stp* deletion had a more pronounced impact on the cell wall than deletion of *stk*. Most strikingly, in the Δ*stp* mutant, the cell wall was thicker and incorporated higher amounts of incomplete muropeptides. The thick cell wall phenotype of Δ*stp* was previously reported for several MRSA strains such as N315^10^, MW2^22^ and a clinical USA300 isolate^17^. The molecular mechanisms leading to this phenotype seem to be manifold as mutations in multiple regulatory pathways such as *vraSR*^34^, *graSR*^34^, *walKR*^34^, and *stk*/*stp*^17^ and lack of nutrients like glycine depletion^35^ have been linked to this phenotype. However, the exact mechanism remains unclear.

In our study, we determined the cell wall composition of *S. aureus* wild type and Δ*stk*, Δ*stp* and Δ*stk*Δ*stp* mutants, which revealed that Δ*stp* incorporated higher amounts of incomplete muropeptides. These incomplete muropeptides had non-glycine, monoglycine and monoalanine interpeptide bridges in the cell wall of stationary phase cells. Interestingly, a similar unexpected cell wall phenotype was observed by Monteiro in a *S. aureus* mutant strain expressing FemX with a C-terminal fusion of GFP. This FemX-GFP mutant strain shows a decreased FemX enzyme activity leading to an altered cell wall composition with an increase of incomplete muropeptides and decrease of pentaglycine-containing muropeptides^36^. FemX catalyzes the introduction of the first glycine of the pentaglycine cross bridge to lipid II. FemX deletion mutants are not viable^37^, therefore the FemX-GFP mutant has to exert residual enzymatic activity. A conditional *femX* repression leads to accumulation of less-crosslinked, unmodified muropeptides^38^. In contrast to FemX, the subsequent enzymes FemA or FemB are non-essential. A *femA* and *femB* deletion led to accumulation of monoglycine or triglycine muropeptides, respectively^39,40^ which decreased the interpeptide cross-linking in the PGN sacculus as compared to wild type cells^41^. Surprisingly, the accumulation of incomplete muropeptides in the *stp* deletion strain did not affect the amount of interpeptide cross-linking. However, the observed structural changes within the interpeptide bridges are a plausible explanation why the *stp* mutant was less susceptible to lysostaphin. As lysostaphin cuts the pentaglycine bridge between the third and the fourth glycine^42^, changes in the amino acid composition affect the cleavage of the interpeptide bridge. Importantly, we showed that Stk phosphorylates and Stp dephosphorylates FemX. Mass spectrometric analysis identified Thr32, Thr36 and Ser415 of FemX as phosphoacceptors. Surprisingly, Stk interacted with FemA and FemB in bacterial two-hybrid studies, but was unable to phosphorylate them *in vitro*. FemA-FemB interaction as well as homodimerization of both proteins were consistent with previous results^43^. Unlike FemA/B proteins, FemX interacted neither with Stk nor with FemA/B proteins nor with itself in the bacterial two-hybrid studies. Despite this missing direct interaction between Stk and FemX in the bacterial two-hybrid studies, the *in vitro* kinase/phosphatase assay suggests an interaction of Stk/Stp with FemX. We hypothesize that regulation of FemX activity by Stk/Stp is the reason for the altered muropeptide composition in the *stp* mutant strain. A realistic scenario could be a phosphorylation-mediated inhibition of FemX activity, which reduces the following cell wall synthesis steps.

Due to the Stk-FemX/A/B interaction profile, we were interested in potential interaction partners of Stk in cell wall synthesis and cell division. In our two-hybrid interaction studies, Stk also interacted with cell wall synthesis enzymes (MurG, FemA/B), PGN cross-linking enzymes (Pbp1-3, RodA, FtsW), as well as the cell wall hydrolase LytH and the monofunctional transglycosylase Mgt. The amidase LytH is predicted in the database aureowiki to be N-terminally anchored in the membrane where Stk could interact with LytH. Taken together this indicates a Stk-dependent regulation of cell wall remodelling. This is in line with observations in other bacteria in which Stk homologs interact with and phosphorylate penicillin-binding proteins like PBP2x^28^, PBP2^32^ and PBPA^44^. The interactions of Stk with Pbp1-3, RodA, FtsW and septal cell division proteins (DivIB, DivIC, EzrA) fit well to the septal localization of Stk^9^. The current model of Stk activation implies that the PASTA domains mediate the septal localization^9^ and induce Stk dimerization^45^. In line with this model, we confirmed Stk dimerization by use of the bacterial two-hybrid system. Following dimerization, *S. aureus* Stk, and also its homologs in other pathogens, are autophosphorylated as has been proven *in vitro*^10,12^. Dephosphorylation of Stk is mediated by direct interaction with the cognate phosphatase Stp *in vitro*. In contrast to Stk, Stp did not interact with any other tested protein in our bacterial two-hybrid studies except for the weak interaction with Stk. Nevertheless, the *in vitro* dephosphorylation assay indicate a direct interaction of Stp with Stk and FemX. Probably, the missing Stp-interactions in bacterial two-hybrid is due to technical reasons, as potential targets of Stp are not phosphorylated under the tested conditions. Furthermore, it should be noted that this bacterial two-hybrid analysis was done in a heterologous expression system, which may influence membrane integration and processing of proteins.

Our metabolome data add further insights into a phosphorylation-dependent regulation of PGN precursor synthesis enzymes by Stk/Stp. Both Stk and Stp had the strongest effects on activity of MurC and MurE/F, considering the increase or decrease of the respective metabolites in the mutant strains. In spite of metabolic changes, Stk and Stp did not interact with the cytosolic cell wall synthesis enzymes (GlmS/M/U, MurA-F) in our bacterial two-hybrid studies. Nevertheless, *in vivo* serine and threonine phosphorylation sites of staphylococcal GlmM^46^ and MurD^47^ clearly indicate a regulation of these enzymes by phosphorylation. Furthermore, other studies showed that the Stk homologs in streptococci and mycobacteria regulate the cell wall synthesis enzymes GlmM^26^, GlmU^48^ and MurC^27^ and interact with the Mur synthetases MurC-F^49^. Moreover, in *Mycobacterium tuberculosis* the activity of these cytosolic cell wall synthesis enzymes is regulated by the Stk homolog PknB through phosphorylation of co-factors like CwlM and Wag31^50,51^. Interestingly, Stk interacted with the membrane-located cell wall synthesis enzyme MurG but not with MraY. Nevertheless, the detected *in vivo* phosphorylation site of MraY^47^ and a decreased tunicamycin sensitivity of the mutant strains indicate Stk-/Stp-dependent regulation. All these data support the idea that Stk/Stp regulates cell wall synthesis and cell division at multiple layers. However, Stk/Stp dependent regulation does not act as on/off system but rather fine-tunes activity of components of the cell wall synthesis and cell division machinery to ensure efficient cell wall synthesis.

**Figure 5:**
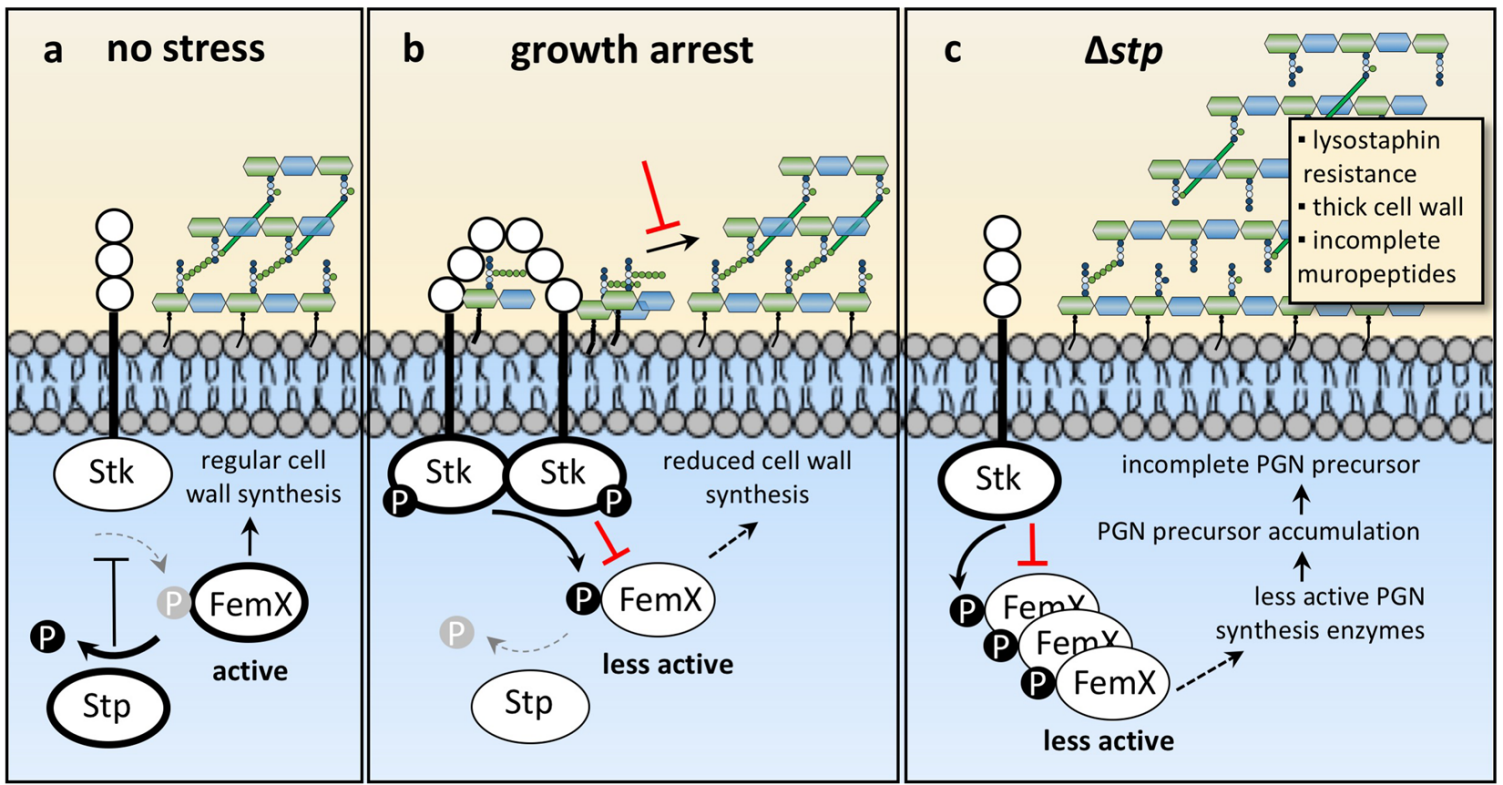
Model for Stk-/Stp-dependent regulation of cell wall synthesis protein FemX. (**a**) During regular growth, the kinase Stk is not phosphorylated and less active. Hence, the phosphatase Stp dephosphorylates the Stk-target FemX and other cell wall synthesis enzymes. (**b**) Following cell growth arrest due to nutrient depletion, accumulated pentaglycine-lipid II activates Stk through PASTA domain-dependent binding^9^. Active Stk phosphorylates FemX and other cell wall synthesis enzymes, leading to downregulation of cell wall synthesis. (**c**) In the *stp* deletion strain, phosphorylated Stk-target proteins like FemX cannot be reversed by dephosphorylation and in consequence accumulate in the cell. This increases the concentration of PGN precursors and incomplete muropeptides are incorporated into the cell wall, resulting in a thicker cell wall and reduced lysostaphin sensitivity.

In our proposed model (Fig. 5), Stk is activated through PASTA domain-dependent binding of accumulated pentaglycine-lipid II^9^ during cell wall stress or growth arrest. Then, active Stk phosphorylates FemX and other cell wall synthesis enzymes. This leads to a reduction of cell wall synthesis and could be a physiological feedback loop for cell wall synthesis. The phosphatase Stp activates cell wall synthesis enzymes like FemX through dephosphorylation of Stk-mediated phosphorylation sites (Fig. 5a,b). This model is supported by the physiological changes observed in the *stp* mutant. The Stp-deficient cells are not able to dephosphorylate FemX, resulting in permanently phosphorylated FemX. In consequence, this less active FemX reduces pentaglycine interpeptide bridge formation of lipid II which results in the incorporation of incomplete muropeptides into the PGN sacculus. The cells likely compensate for the altered PGN composition by increasing cell wall thickness (Fig. 5c). Based on these results, phosphatase inhibitors may be developed as promising antibacterial agents that induce deregulation of bacterial cell wall metabolism and, in combination with cell wall-active antibiotics, could overcome the current shortage in the treatment of *S. aureus* and other bacteria.

## Methods

### Strains, media and growth conditions

The strains and plasmids used in this study are listed in Tables S8–12. *S. aureus* NewmanHG (NewHG)^52^ strains were grown in Tryptic soy broth (TSB, HiMedia, India) and *E. coli* strains in lysogenic broth (LB). The bacteria were shaken at 180 rpm and 37 °C, unless indicated otherwise.

### Construction of *S. aureus* NewmanHG Δ*stk*, Δ*stp* and Δ*stk*Δ*stp* strains

The *S. aureus* NewmanHG mutant strains lacking *stk*, *stp* or both *stk* and *stp* genes were constructed as previously described for *S. aureus* 8325 Δ*pknB*^12^. Briefly, fragments upstream and downstream of *stk*, *stp* and *stk/stp* were amplified by PCR using listed primers (Table S11). The erythromycin resistance cassette (*ermB*, 1346 bp) was amplified from the pEC1^53^ using *ermB* primers (Table S11). The fragments were cloned into the pGEM-T vector (Promega, Mannheim, Germany) and then cut out with the corresponding restriction enzymes and cloned into the temperature-sensitive shuttle pBT2. These vector constructs were electroporated into *S. aureus* RN4220, transduced to *S. aureus* NewmanHG via phage Φ85, and chromosomally integrated after temperature shift. The deletions were verified by PCR using gene-specific primers and *S. aureus* Genotyping Kit 2.0 (Alere, Jena, Germany).

### Transmission electron microscopy

For transmission electron microscopy (TEM), the strains were grown in 20 ml TSB medium at 37 °C. The optical density (OD_600_) of the culture was measured at 600 nm. 15 ml of late logarithmic (OD_600_ 2) and 1 ml of stationary phase culture (overnight, OD_600_ 10) were centrifuged (10 min, 8,000 × g) and the cell pellet was fixed in 2.5% glutaraldehyde buffered in cacodylate buffer for 30 min on ice. Further electron microscopy sample preparation processing was conducted according to Prüfert *et al*.^54^. Electron micrographs were recorded at 200 kV acceleration voltage on a JEM-2100 electron microscope (JEOL, Freising, Germany) with the digital camera TemCam F416 (TVIPS, Gauting, Germany). The mean cell diameter (CD) and the cell wall thickness (CWT) of *S. aureus* wt and mutant cells based on the diameter of the 20 largest cells out of 100 cells per strain at five different spots on the cell wall. We chose the 20 largest cells without septa to ensure that all cells are in the same stage of the cell cycle. Significance values of CD and CWT were calculated using a two-tailed unpaired student t-test (*p < 0.05, **p < 0.01, ***p < 0.001).

### Analysis of muropeptide profiles

The strains were grown at 37 °C in 20 ml TSB medium to an OD_600_ of 2 (late logarithmic growth phase) and overnight (OD_600_ 9 to 12, stationary growth phase). Four ml of logarithmic phase culture or 2 ml of stationary phase culture were centrifuged (20 min, 5,000 × g) and the cell pellets were stored at −80 °C. PGN was isolated as previously described^55^ with the following exceptions: (1) For digestion into muropeptides 100 µl of the resuspended PGN (OD_600_ = 3) were incubated with 20 µl mutanolysin (2,000 U/ml) for 21 h at 37 °C on a magnetic stirrer. (2) For UPLC analysis a 100 mM sodium phosphate buffer with pH 2.5 (buffer A), instead of TFA, was used in a linear gradient from 95% buffer A and 5% methanol to 70% buffer A and 30% methanol in 70 min with a flow rate of 0.167 ml/min at 52 °C column temperature. UPLC-MS was performed exactly as describe previously^55^. Muropeptides were determined by their retention times and their masses. The amounts given are the relative abundances of the respective peak area as percentage of all muropeptide peaks. The mean and standard deviation was calculated as integral under the peak in comparison to integral of the whole chromatogram. The muropeptide interpeptide cross-linking was calculated according to following formula^5^.

$${\rm{cross}}\,\mathrm{linking}\,\,[ \% ]=1/2\,{\rm{dimer}}+2/3\,{\rm{trimer}}+3/4\,{\rm{tetramer}}+9/10\,{\rm{oligomer}}$$

### Lysostaphin susceptibility assay

The strains were grown at 37 °C TSB medium to an OD_600_ of 2 (late logarithmic growth phase) and overnight (OD_600_ 5 to 7, stationary growth phase). The cultures of *S. aureus* strains were adjusted to OD_600_ of 2, and 1 ml cell suspensions were pelleted. After washing once with 1 ml of lysostaphin buffer (20 mM Tris-HCl pH 7, 150 mM NaCl, 1 mM EDTA), the pellets were then resuspended in 1 ml lysostaphin buffer. One hundred µl of 1 µg/ml lysostaphin (Bharat Biotech, Hyderabad, India) was added to every 100 µl of each bacterial suspension in a 96-well plate and incubated at 37 °C in a microplate reader (TECAN, Maennedorf, Switzerland). The OD_600_ of each culture was read every 5 minutes and plotted as a percentage of the initial reading, which was set to 100% and 0 minutes. Significance values of lysostaphin susceptibility were calculated using a two-tailed unpaired student t-test (*p < 0.05, **p < 0.01, ***p < 0.001).

### Determination of intracellular metabolites

Cell wall precursor concentrations were determined according to a recently published protocol^11,56^. Briefly, 20 OD_600_ units of the 100 ml TSB cell culture broth of logarithmic (OD_600_ 2) and stationary phase (OD_600_ 9 to 12) cells were separated by fast filtration, and the cells were washed with 15 ml of 0.6% ice-cold sodium chloride solution. The filter was immediately transferred to a tube filled with 5 ml of ice-cold extraction solution (60% [wt/vol] ethanol) and quickly frozen in liquid nitrogen. The frozen sample was stored at −80 °C. Every following extraction step was carried out on ice. The bacteria were washed off the filter by shaking and vortexing in extraction solution. After centrifugation (10 min, 10,000 rpm at 4 °C) the supernatant was transferred into a 50-ml tube containing glass beads with a diameter of 0.1 μm (Sigma-Aldrich, Taufkirchen, Germany). After washing the filter with 5 ml ultrapure water (HPLC grade) and adding the internal standard for LC-MS analysis (5 nmol camphorsulfonic acid) to the combined cell suspensions, cell disruption was performed by using a FastPrep-24 instrument (MP Biomedicals, Eschwege, Germany) twice for 45 s each 6.0 m/s. Cell lysate was separated from cell debris by centrifugation (10 min, 12,000 rpm at 4 °C) and the supernatant was transferred into a 50-ml tube. The cell debris was washed again with 5 ml ultrapure water and a second cell disruption was performed by FastPrep. The supernatants were combined, diluted with 20 ml ice cold water (HPLC-MS grade) and stored at −80 °C before lyophilization. The lyophilized samples were dissolved in 100 µl water and centrifuged (3 min at room temperature). The supernatant was transferred into a glass vial. Ion pairing LC-MS measurement and data analysis was performed as previously described^57^. Changes in PGN precursor levels between wild type and mutant strains were compared using the non-parametric unpaired Mann-Whitney test. Differences were considered significant when p-value < 0.05.

### Bacterial two-hybrid analysis to test protein-protein interaction

Strains and plasmids used in this work are listed in Tables S9 and S12. The bait vectors p25N (pKNT25) and p25C (pKT25), the prey vectors p18C (pUT18C) and p18N (pUT18), the control plasmids pKT25-*zip* and pUT18C-*zip* and the Δ*cya* reporter strain *E. coli* BTH101 were obtained from EuroMedex (Stouffelweyersheim, France). The genes encoding the protein of interests are amplified from *S. aureus* NewmanHG genomic DNA by PCR and fused in-frame to the 3′ (p18N or p25N) or 5′ end (p18C or p25C) of the *cyaA* gene fragment of the appropriate vectors. *stk, stp* and *femX/A/B* genes were cloned into the bait vector p25C and the prey vector p18C. PGN synthesis- and cell wall division genes were cloned into the p18C vector or were a kind gift from S. Foster (Sheffield, UK)^58^ or D. Lopez (National Centre for Biotechnology, Madrid, Spain) (Table S9).

The recombinant plasmids encoding T25- or T18- hybrid proteins are transformed into competent *E. coli* BTH101 reporter cells. Transformants are plated on selective plates (LB agar with 50 µg/ml kanamycin, 100 µg/ml ampicillin and streptomycin) and incubated at 30 °C for up to two days. Five to 10 colonies were combined and incubated overnight in LB media with the appropriated antibiotics at 30 °C. Two µl overnight culture was spotted on indicator plates (LB agar with appropriated antibiotics, 40 µg/ml X-gal, 0.5 mM IPTG) using a replica plater (Sigma-Aldrich, Taufkrichen, Germany) and incubated for 5 days at 18 °C. As a negative control each bait vector was tested against an empty prey vector and *vice versa*. The control plasmids pKT25-*zip*/pUT18-*zip* were used as a positive control. Each plasmid combination was tested at least three times on indicator plates. The classification of protein-protein interactions is based on the time point when the colonies change their colour. The positive control (pKT25-*zip*/pUT18-*zip*) and positive interactions changed the colour to blue after 2 to 4 days. No interactions showed no colour change after 5 days. If a slight colour change was visible after 5 days, then we interpreted this interaction as weak and this interaction should be characterized with another method. We stopped the assay after 5 days, when nearly all combinations as well as the negative controls showed a slight background reaction. To check the specificity of the interaction, every vector was also tested with their empty partner vector p18C or p25C vector. Moreover, we quantified the β-galactosidase activity of selected protein-protein interactions based on spot assay at Fig. 4a (Fig. S13).

### Cloning, expression and purification of recombinant proteins

The recombinant N-terminal polyhistidine-tagged proteins Stp, Stk_KD_, FemX, FemA and FemB were cloned and purified as previously reported^12^. Briefly, the coding sequence of the genes and the coding sequence of the Stk kinase domain (amino acid 1 to 291) (Stk_KD_)^33^ were amplified by PCR using genomic DNA of *S. aureus* NewmanHG and primers listed in Table S11. The gene fragments were restricted with appropriate enzymes and ligated into the pET28a vector (Novagen, Wisconsin, USA). This vector was transformed into *E. coli* BL21 (DE3) cells for protein expression. For expression of recombinant proteins, one liter of *E. coli* cultures were grown at 37 °C until an OD_600_ of 0.5 to 0.7 was reached. After adding 0.5 mM final concentration of IPTG, the cells were incubated overnight at 18 °C. The cells were harvested (15 min, 4,000 × g, 4 °C) and the cell pellet was stored at −20 °C. The proteins were purified under native conditions by affinity chromatography on Proteino Ni-TED columns by following the manufacturer’s instructions (Macherey-Nagel, Düren, Germany). Briefly, the cell pellet was resuspended in 10 ml lysis buffer (50 mM NaH_2_PO_4_, 300 mM NaCl, 50 µg/ml DNase, 100 µg/ml lysozyme, 0.02% Calbiochem Protease Inhibitor Cocktail Set 1 [Merck, Darmstadt, Germany]) and incubated 30 min on ice. The cells were sonicated (5 × 45 s) and centrifuged (30 min, 8,000 × g, 4 °C). The supernatant was loaded on the Ni-TED columns, and protein purification was performed following the manufacturer’s instructions. The eluates were dialyzed in 50 mM Tris-HCl pH 7.5, 300 mM NaCl and concentrated using Amicon Ultra filter (Merck, Darmstadt, Germany). Protein concentration was determined by Advanced Protein Assay (Cytoskeleton, Denver, USA).

### *In vitro* phosphorylation/dephosphorylation assay

One µg recombinant proteins (FemX, FemA or FemB) were incubated with 1 µg Stk kinase domain (Stk_KD_) in kinase buffer (50 mM Tris-HCl pH 7.5, 3 mM MgCl_2_, 3 mM MnCl_2_) with or without 20 mM ATP for 1 h at 37 °C. For the dephosphorlyation reaction, 1 µg Stp was added to the reaction mixture. Each reaction was stopped by addition of 5 x SDS sample buffer and heating for 5 min 95 °C. The phosphorylated proteins were detected by Western blotting using an anti-phosphothreonine antibody (#9381 S, Cell Signaling, Frankfurt, Germany). SDS-PAGE was performed and the proteins were blotted on a nitrocellulose membrane using a semi-dry blotting technique. The blot was blocked in 5% albumin (Sigma-Aldrich, Taufkirchen, Germany) in TBS (10 mM Tris-HCl pH 7.5, 0.9% NaCl) for 2 h and then incubated with anti-phosphothreonine antibody (1:5,000, 1% albumin/TBS-T) overnight at 4 °C. After washing the blot three times in TBS-T (TBS + 0.05% Tween 20) for 15 min, the blot was incubated with anti-rabbit-HRP antibody (#L3012, SAB, USA) (1:10,000 in TBS) for 2 h. After three additional TBS-T washing steps, the blot was incubated with ECL substrate for 2 min. Phosphorylated proteins were visualized using ImageQuant LAS 4000 imaging system (GE Healtcare, Munich, Germany).

### Mass Spectrometry

In order to characterize phosphorylation sites of the FemX/A/B proteins, we performed mass spectrometry. The *in vitro* phosphorylation/dephosphorlyation reaction was stopped by heating the sample for 5 min at 95 °C. The sample was then reduced for 30 min with 1 mM dithiothreitol and alkylated for 30 min in the dark with 10 mM iodoacetamide. After adding 5x SDS sample buffer and heating the sample for 5 min at 95 °C, SDS-PAGE was performed. The corresponding protein bands of the Coomassie-stained SDS-gel were cut out and destained with 30% acetonitrile in 0.1 M NH_4_HCO_3_ (pH 8), shrunk with 100% acetonitrile and dried in a vacuum concentrator (Concentrator 5301, Eppendorf, Germany). Digests were performed with 0.1 µg elastase per gel band overnight at 37 °C in 0.1 M NH_4_HCO_3_ (pH 8). After removing the supernatant, peptides were extracted from the gel slices with 5% formic acid, and extracted peptides were pooled with the supernatant.

NanoLC-MS/MS analyses were performed on an Orbitrap Fusion (Thermo Scientific) equipped with a PicoView Ion Source (New Objective) and coupled to an EASY-nLC 1000 (Thermo Scientific). Peptides were loaded on capillary columns (PicoFrit, 30 cm × 150 µm ID, New Objective) self-packed with ReproSil-Pur 120 C18-AQ, 1.9 µm (Dr. Maisch, Ammerbuch-Entringen, Germany) and separated with a 30-minute linear gradient from 3% to 30% acetonitrile and 0.1% formic acid and a flow rate of 500 nl/min.

Both MS and MS/MS scans were acquired in the Orbitrap analyser with a resolution of 60,000 for MS scans and 15,000 for MS/MS scans. HCD fragmentation with 35% normalized collision energy was applied. Triply and higher charged precursors were also selected for ETD fragmentation using EasyETD option. A Top Speed data-dependent MS/MS method with a fixed cycle time of 3 seconds was used. Dynamic exclusion was applied with a repeat count of 1 and an exclusion duration of 120 seconds; singly charged precursors were excluded from selection. Minimum signal threshold for precursor selection was set to 50,000. Predictive AGC was used with AGC a target value of 5e5 for MS scans and 5e4 for MS/MS scans. EASY-IC was used for internal calibration.

Data analysis was performed with PEAKS Studio 8.0 with the following parameters: parent mass error tolerance: 8 ppm, fragment mass error tolerance: 0.02 Da, enzyme: none, variable modifications: pyro-Glu (N-term. Q), oxidation (M), carbamidomethyl (C), phosphorylation (STY), acetylation (protein N-term.), max. variable modifications per peptide: 3. Database search was performed against a custom database containing the sequence of the FemX/A/B, Stk and Stp. Results were filtered to 1% FDR on the peptide level.

### Antibiotic susceptibility testing

Minimal inhibitory concentrations (MIC) of all listed antibiotics (Fig. S6) were determined by microdilution according to the recommendations of the Clinical and Laboratory Standard Institute guidelines^59^. The MICs were determined in 96-well microtiter plates using a final volume of 200 µl TSB media, starting with an initial inoculum of OD_600_ 0.05 per well. Plates were incubated for 24 h at 37 °C.

## Electronic supplementary material

Supplemetary information
