# Supplementary material for: The serine/threonine kinase Stk and the phosphatase Stp regulate cell wall synthesis in Staphylococcus aureus
Source: Sci Rep. 2018 Sep 12;8:13693. doi: 10.1038/s41598-018-32109-7 (PMC6135852; doi:10.1038/s41598-018-32109-7)
Supplement: Supplementary file 1 — Supplemetary information [file 41598_2018_32109_MOESM1_ESM.pdf]

Supplementary Information

The serine/threonine kinase Stk and the phosphatase Stp regulate cell wall synthesis in *Staphylococcus aureus*

Marcel Jarick[1], Ute Bertsche[2,3], Mark Stahl[4], Daniel Schultz[5], Karen Methling[5], Michael Lalk[5], Christian Stigloher[6], Mirco Steger[7], Andreas Schlosser[7], Knut Ohlsen[1]*

1 University of Würzburg, Institute for Molecular Infection Biology, Würzburg, Germany

2 University of Tübingen, IMIT - Infection biology, Tübingen, Germany

3 University of Hohenheim, Core Facility - Module 1 Mass Spectrometry Unit, Stuttgart, Germany

4 University of Tübingen, Center for Plant Molecular Biology, Tübingen, Germany

5 University of Greifswald, Institute of Biochemistry, Greifswald, Germany

6 University of Würzburg, Imaging Core Facility - Biocenter, Würzburg, Germany

7 University of Würzburg, Rudolf Virchow Center for Experimental Biomedicine, Würzburg, Germany

*Correspnding author: knut.ohlsen@uni-wuerzburg.de

Contact Inforamtion:
PD Dr. Knut Ohlsen, University Würzburg, Institut for Molecular Infection Biology, Josef-Schneider-Straße 2, 97080 Würzburg

Tel: ++49-(0)931-31 82155
E-Mail: knut.ohlsen@uni-wuerzburg.de

Supplementary Information

Figure S1. Cell wall phenotype of *S. aureus* NewmanHG wt, Δ*stk*, Δ*stp* and Δ*stk*Δ*stp* strains at logarithmic growth phase.

Table S2. Muropeptide of *S. aure*us analyzed by UPLC-MS.

Figure S3. PGN precursor concentration in *S. aureus* wild type and mutant strains.

Figure S4. Protein interaction network of Stk, Stp and FemX/A/B.

Figure S5a-d. Identification of *in vitro* phosphorylation site of FemX.

Figure S5e. MS/MS spectra of tryptic FemX peptides containing the Stk phosphorylation sites.

Figure S5f-h. Identification of *in vitro* phosphorylation site of FemA.

Figure S5i-k. Identification of *in vitro* phosphorylation site of FemB.

Table S6. Minimal inhibitory concentration (MIC) of cell wall-active antibiotics for *S. aureus* NewmanHG wt and mutant strains.

Figure S7. Stk/Stp *in vitro* kinase/phosphatase assay.

Table S8. Bacterial strains used in this study.

Table S9. *E. coli* bacterial two-hybrid strains used in this study.

Table S10. Plasmids used in this study.

Table S11. Primer used in this study.

Table S12. Primer for constructing bacterial two-hybrid vectors.

Figure S13. Quantitative measurement of selected protein interactions.

Supplementary References

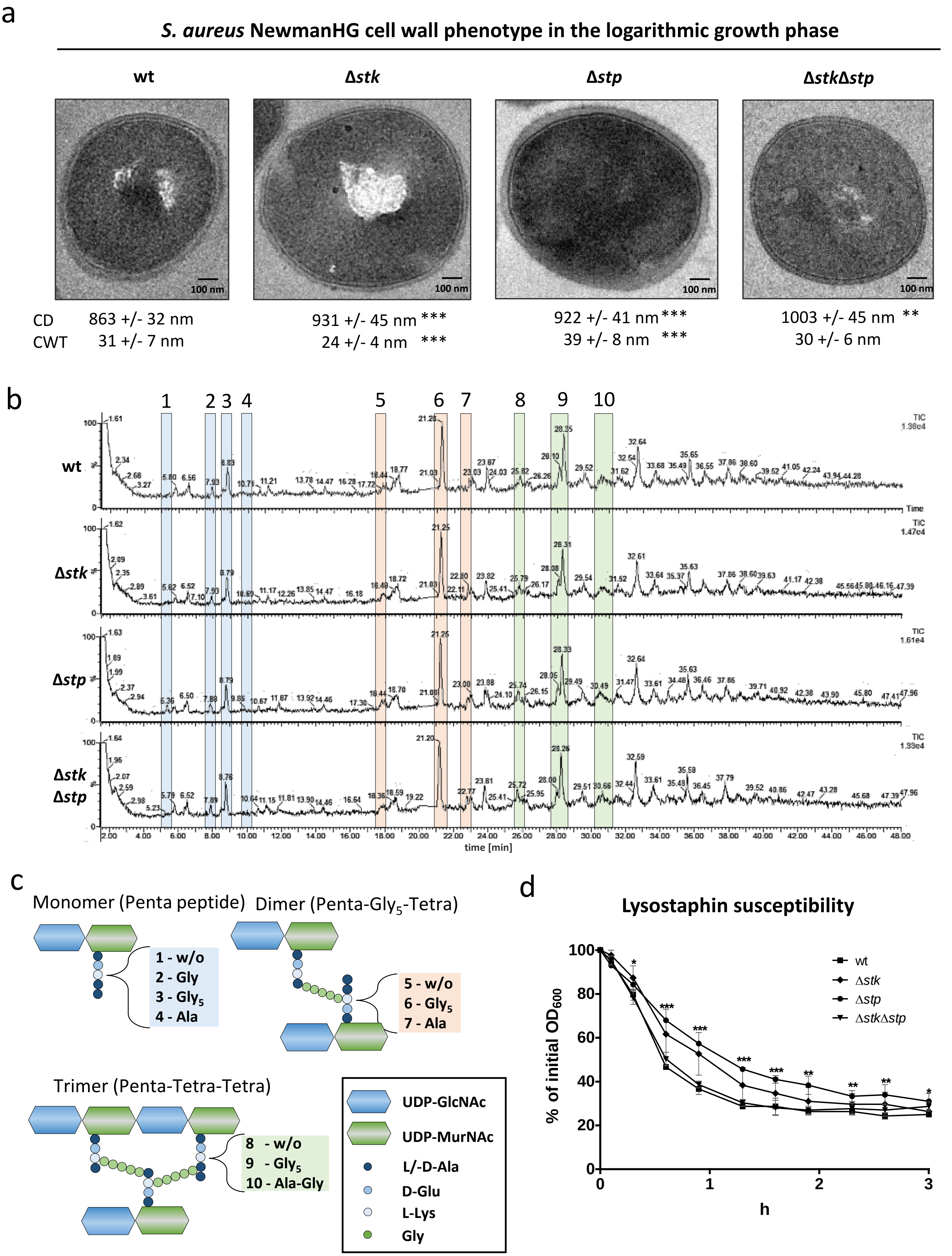
a
S. aureus NewmanHG cell wall phenotype in the logarithmic growth phase
wt
Δstk
Δstp
ΔstkΔstp
100 nm
CD
CWT
863 +/- 32 nm
31 +/- 7 nm
931 +/- 45 nm ***
24 +/- 4 nm ***
922 +/- 41 nm ***
39 +/- 8 nm ***
1003 +/- 45 nm **
30 +/- 6 nm
b
1 2 3 4 5 6 7 8 9 10
wt
Δstk
Δstp
Δstk Δstp
TIC
time [min]
c
Monomer (Penta peptide)
1 - w/o
2 - Gly
3 - Gly5
4 - Ala
Dimer (Penta-Gly5-Tetra)
5 - w/o
6 - Gly5
7 - Ala
Trimer (Penta-Tetra-Tetra)
8 - w/o
9 - Gly5
10 - Ala-Gly
UDP-GlcNAc
UDP-MurNAc
L/-D-Ala
D-Glu
L-Lys
Gly
d
Lysostaphin susceptibility
% of initial OD600
h
wt
Δstk
Δstp
ΔstkΔstp

Figure S1. Cell wall phenotype of *S. aureus* NewmanHG wt, Δ*stk,* Δ*stp* and Δ*stk*Δ*stp* strains at logarithmic growth phase.

(**a**) Analysis of the cell morphology and the cell wall thickness of *S. aureus* wt and mutant at the same stage in the cell cycle by TEM. The mean cell diameter (CD) and the cell wall thickness (CWT) was assessed based on the diameter of the 20 largest cells out of 100 cells per strain. The CWT was measured at five different points on the cell wall. (**b**) Muropeptide profile of *S. aureus* wt and mutant strains obtained by UPLC-MS. Highlighted muropeptide peaks were identified by MS and their structures are shown (**c**). Masses and retention times of indicated peaks are shown in Fig. S2a. (**d**) Lysostaphin susceptibility of *S. aureus* wt and mutant strains. Data represent percentages of the initial optical density (OD_{600}) over three hours after treatment with 0.5 µg/ml lysostaphin. The graphs show the mean and standard deviation of three independent experiments. Significance values of CD, CWT and lysostaphin susceptibility were calculated using a two-tailed unpaired student t-test (* $p < 0.05$, ** $p < 0.01$, *** $p < 0.001$).

a

peak	structure	t_{min}	$M + H^+$
1	Penta	5.3	968.5
2	Penta-Gly	7.8	1025.5
3	Penta-Gly_5	8.8	1253.5
4	Penta-Ala	10.0	1039.5
5	Penta-Gly_5-Tetra	17.7	2132.0
6	Penta-Gly_5-Tetra-Gly_5	21.1	2417.1
7	Penta-Gly_5-Tetra-Ala	22.6	2203.0
8	Trimer 2x Gly_5	25.9	3295.5
9	Trimer 3x Gly_5	28.0	3580.6
10	Trimer 2x Gly_5 + Ala-Gly	30.5	3423.6

b

		wt		Δ*stk*		Δ*stp*		Δ*stk*Δ*stp*	
peak	structure	mean [%]	STD	mean [%]	STD	mean [%]	STD	mean [%]	STD
1	**Penta**	0.83	0.05	0.76	0.21	**1.98**	0.03	0.67	0.03
2	**Penta-Gly**	0.53	0.14	0.47	0.02	**0.62**	0.04	0.46	0.03
3	Penta-Gly_5	0.60	0.12	0.70	0.01	0.48	0.00	0.68	0.03
4	**Penta-Ala**	0.57	0.07	0.55	0.09	**0.87**	0.14	0.54	0.04
5	**Penta-Gly_5-Tetra**	0.41	0.03	0.38	0.01	**0.59**	0.03	0.39	0.01
6	Penta-Gly_5-Tetra-Gly_5	1.37	0.11	1.35	0.11	1.02	0.05	1.36	0.04
7	Penta-Gly_5-Tetra-Ala	0.98	0.10	1.01	0.19	0.93	0.03	1.28	0.14
8	**Trimer 2x Gly_5**	0.55	0.04	0.47	0.02	**0.82**	0.08	0.52	0.01
9	Trimer 3x Gly_5	1.28	0.06	1.31	0.05	1.04	0.01	1.50	0.33
10	**Trimer 2x Gly_5 + Ala-Gly**	0.62	0.05	0.48	0.10	**0.87**	0.08	0.48	0.12

c

	cross-linked muropetides [%]			
strain	wt	Δ*stk*	Δ*stp*	Δ*stk*Δ*stp*
Mean	73.61	72.76	72.11	72.65
STD	0.66	0.16	0.35	0.40

Table S2. Muropeptide of *S. aure*us analyzed by UPLC-MS.

(**a**) The specific mass (m) and retention time (t_{min}) of the muropeptide of *S. aureus* strains (Fig. 1b + S2a). The structure of the muropeptide of the respective peaks was identified by MS. (**b**) Relative quantitative data of the stationary phase *S. aureus* strains are shown. The concentration of each muropeptide were calculated from the area under the peak in comparison to the whole chromatogram (n=3). (**c**) Muropeptide cross-linking showed no differences between the *S. aureus* strains.

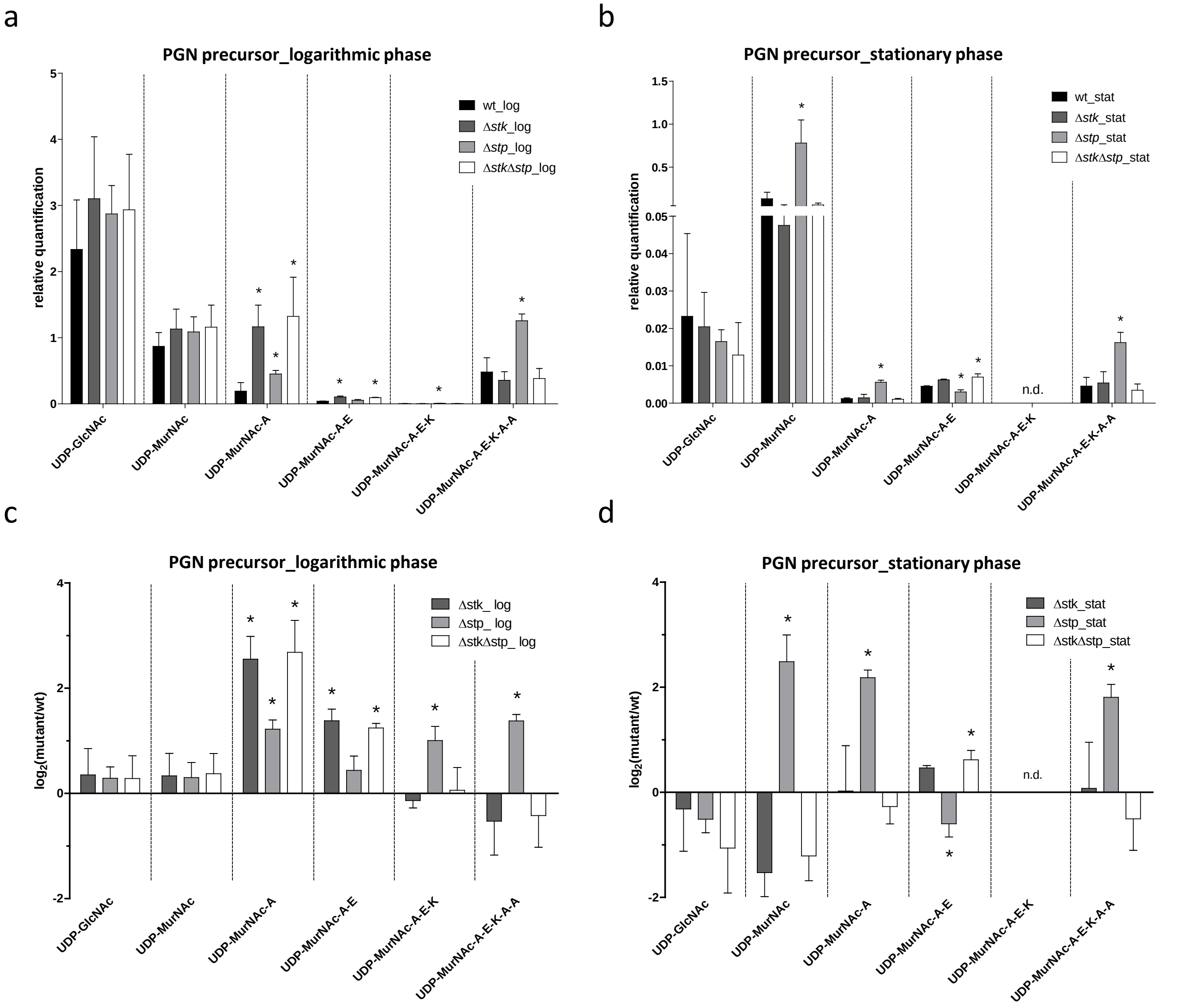

Figure S3. PGN precursor concentration in *S. aureus* wild type and mutant strains.

Relative PGN precursor concentration of *S. aureus* strains at logaritmic- (**a**) and stationary-growth phase (**b**) adjusted to an internal standard (5 nmol camphorsulfonic acid). Graph (**c**) and (**d**) show the same data represented as the $\log_2$-fold changes of Δ*stk*, Δ*stp* and Δ*stk*Δ*stp* mutants compared to wild-type cells at logaritmic- and stationary-growth phase. Significant differences (n=4; non-parametric unpaired Mann-Whitney test; * $p < 0.05$) are marked with asterisks. UDP-MurNAc-A-E-K was not detectable (n.d.) at stationary-growth phase. A alanine, E glutamic acid, K lysine

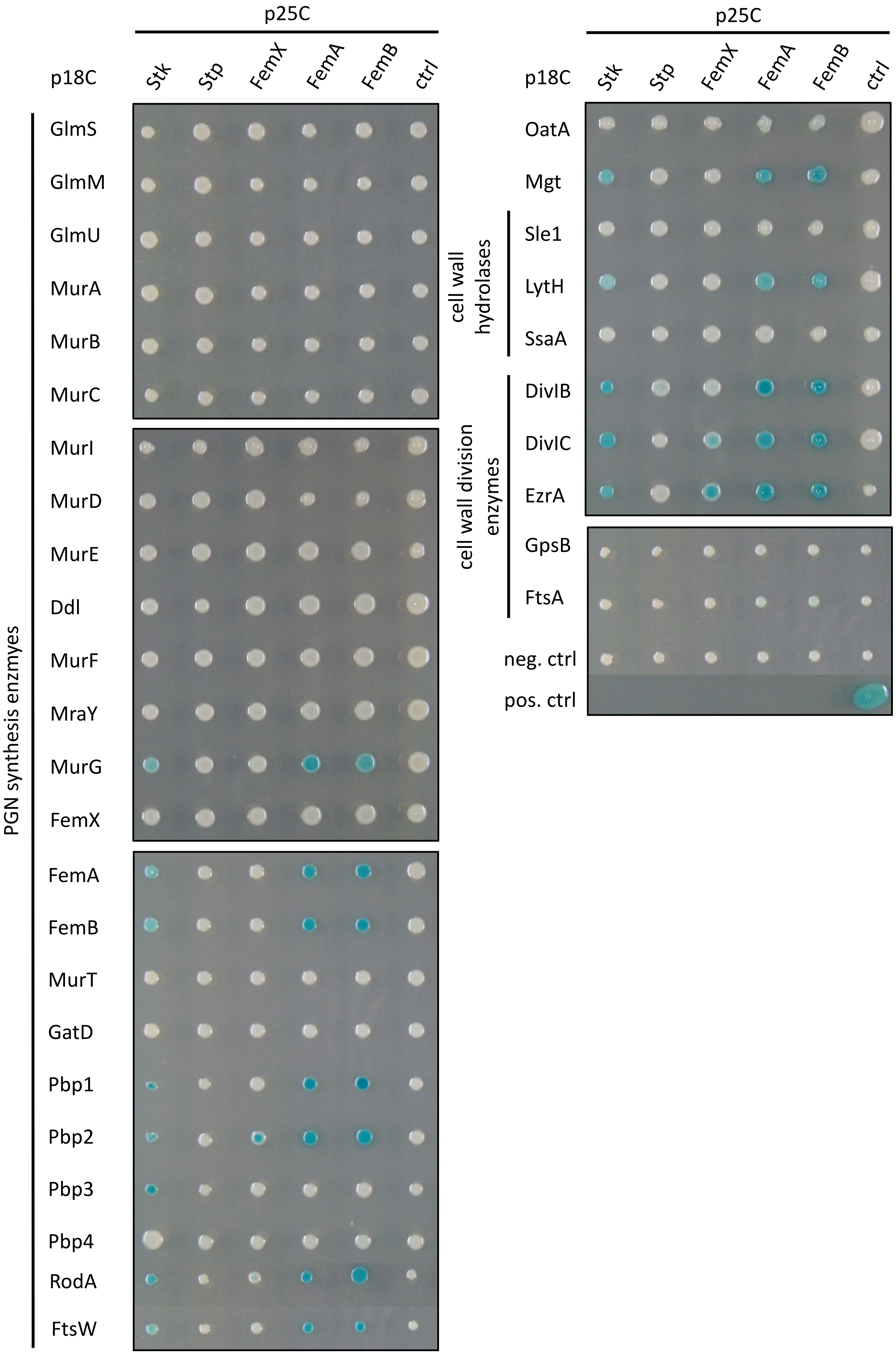
p25C
p18C
Stk
Stp
FemX
FemA
FemB
ctrl
PGN synthesis enzmyes
GlmS
GlmM
GlmU
MurA
MurB
MurC
MurI
MurD
MurE
Ddl
MurF
MraY
MurG
FemX
FemA
FemB
MurT
GatD
Pbp1
Pbp2
Pbp3
Pbp4
RodA
FtsW
p25C
p18C
Stk
Stp
FemX
FemA
FemB
ctrl
OatA
Mgt
cell wall hydrolases
Sle1
LytH
SsaA
cell wall division enzymes
DivIB
DivIC
EzrA
GpsB
FtsA
neg. ctrl
pos. ctrl

Figure S4. Protein interaction network of Stk, Stp and FemX/A/B

The bacterial two-hybrid assay was used to test protein-protein interactions between Stk, Stp, FemX/A/B and cell wall synthesis proteins, cell division proteins as well as cell wall hydrolases. Co-transformants of Δ*cya* mutant *E. coli* BTH101 were spotted onto X-gal indicator plates. The blue color indicated the reconstitution of the functional adenylate cyclase due to the interaction of the fusion proteins. pKT25-*zip*/pUT18-*zip* represented the positive control, whereas empty plasmids p25C and p18C represented negative controls. Every interaction was tested in at least three independent experiments.

a **FemX**, w/o ATP, w/o Stk,w/o Stp

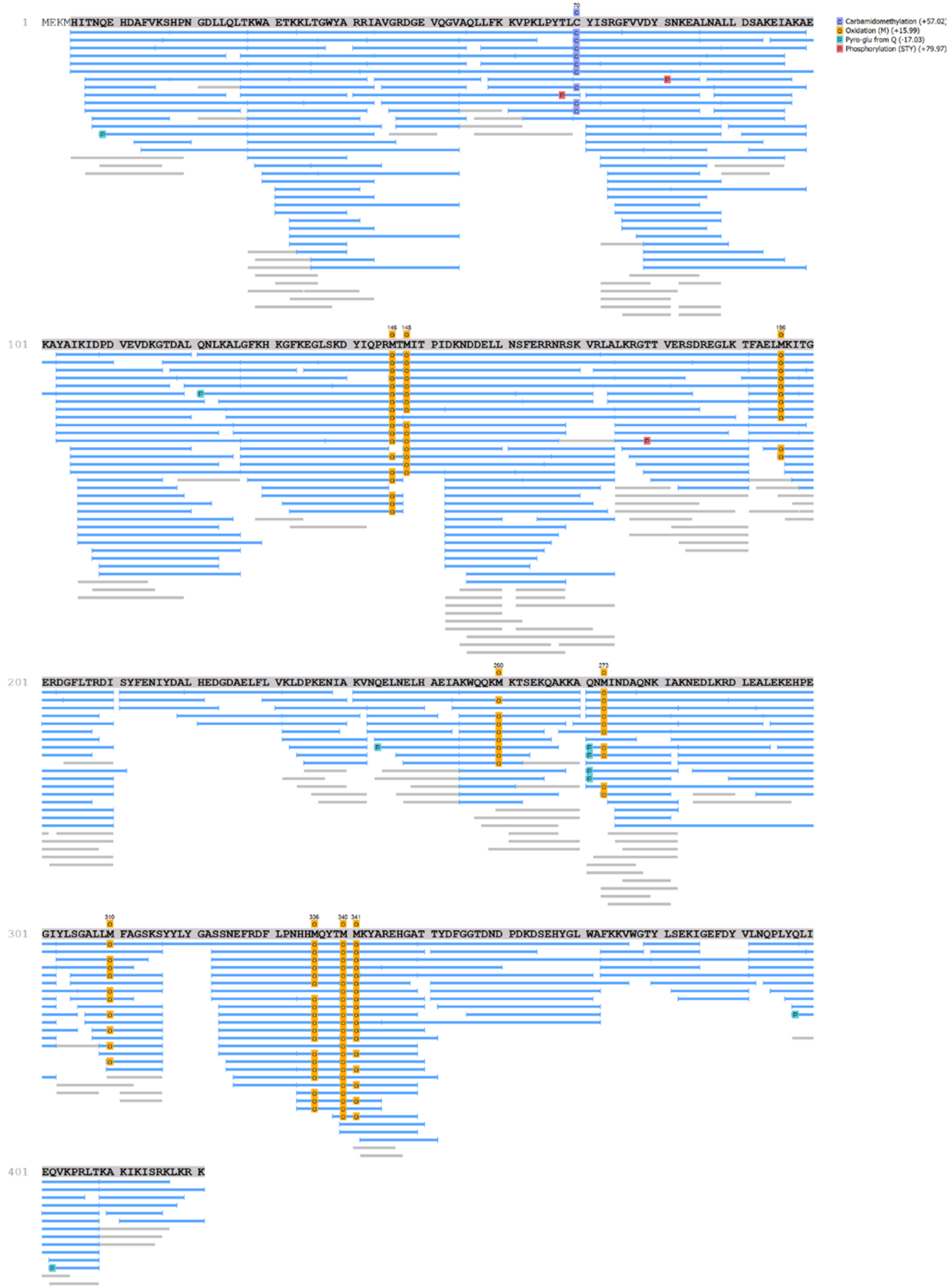

Figure S5a. Identification of *in vitro* phosphorylation site of FemX.
Recombinant FemX were incubated without ATP and Stk_{KD} in kinase buffer for 1 h at 37 °C. After SDS-PAGE, the appropriate FemX protein band was digested with elastase and the peptides were measured by LC-MS/MS. Phosphorylated peptides are marked with red boxes.

b **FemX** + ATP + **Stk**,w/o Stp

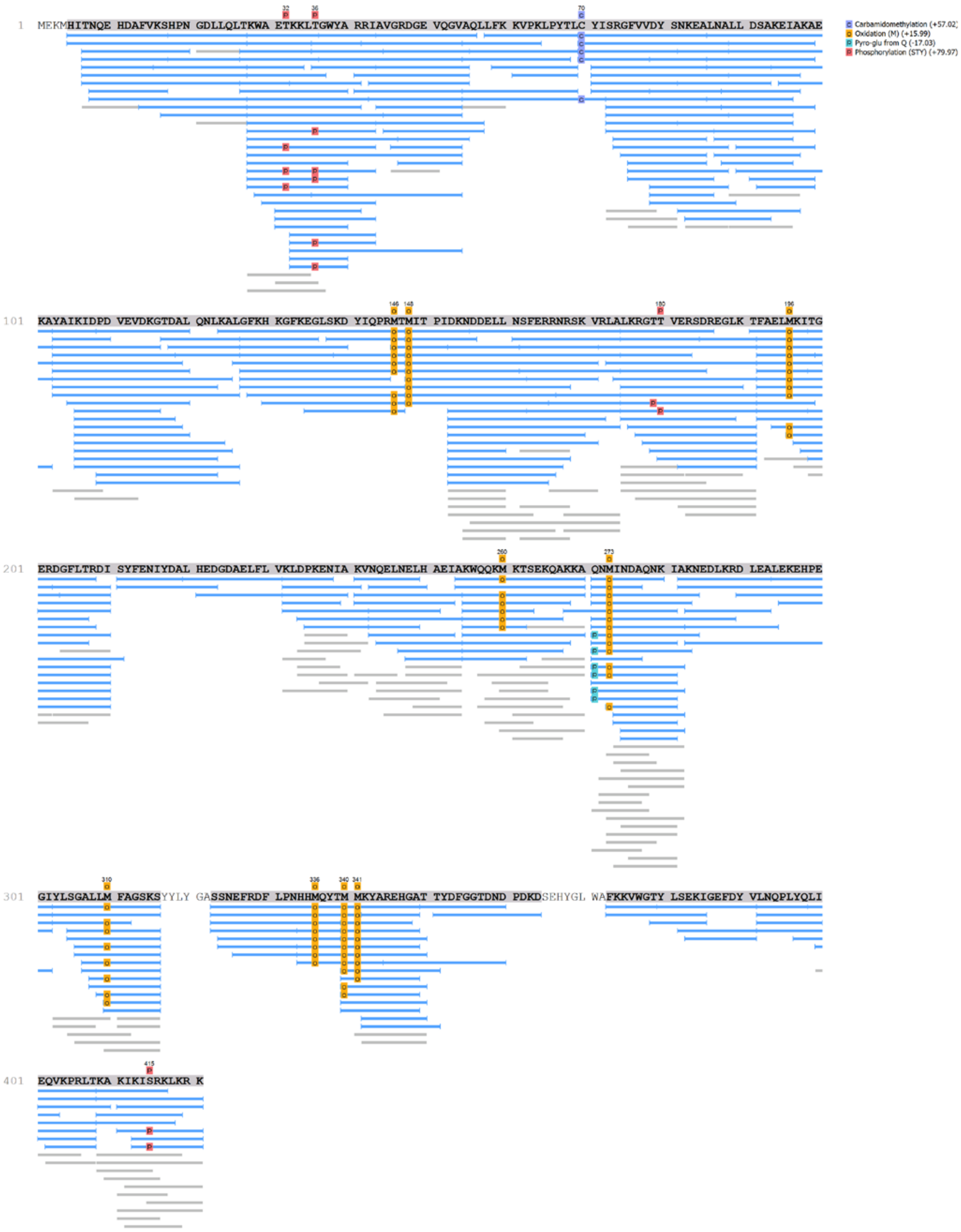

Figure S5b. Identification of *in vitro* phosphorylation site of FemX.
Recombinant FemX were incubated with 20 mM ATP and Stk_{KD} in kinase buffer for 1 h at 37 °C. After SDS-PAGE, the appropriate FemX protein band was digested with elastase and the peptides were measured by LC-MS/MS. Phosphorylated peptides are marked with red boxes.

c **FemX** + ATP + **Stk** + **Stp**

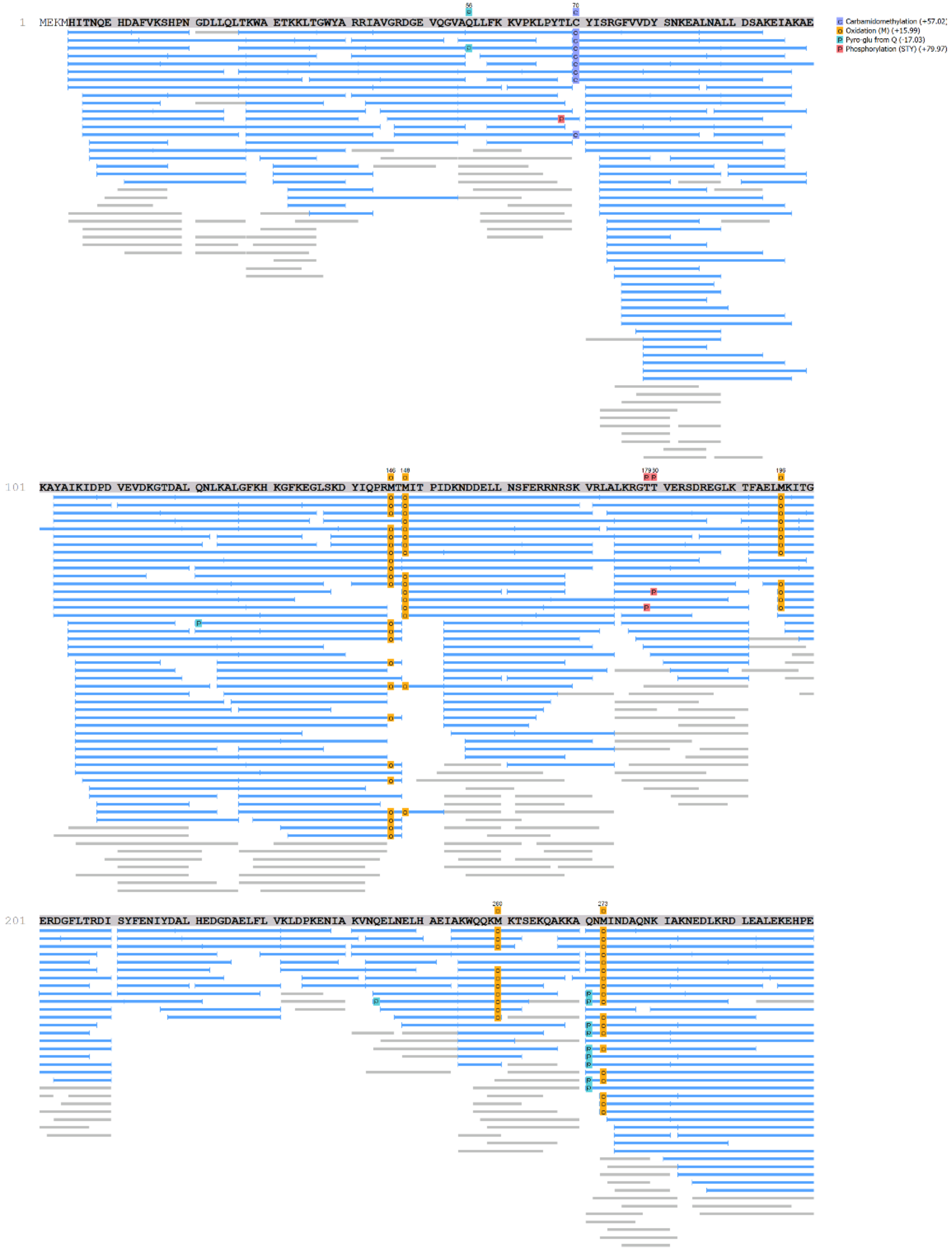

c **FemX** + ATP + **Stk** + **Stp**

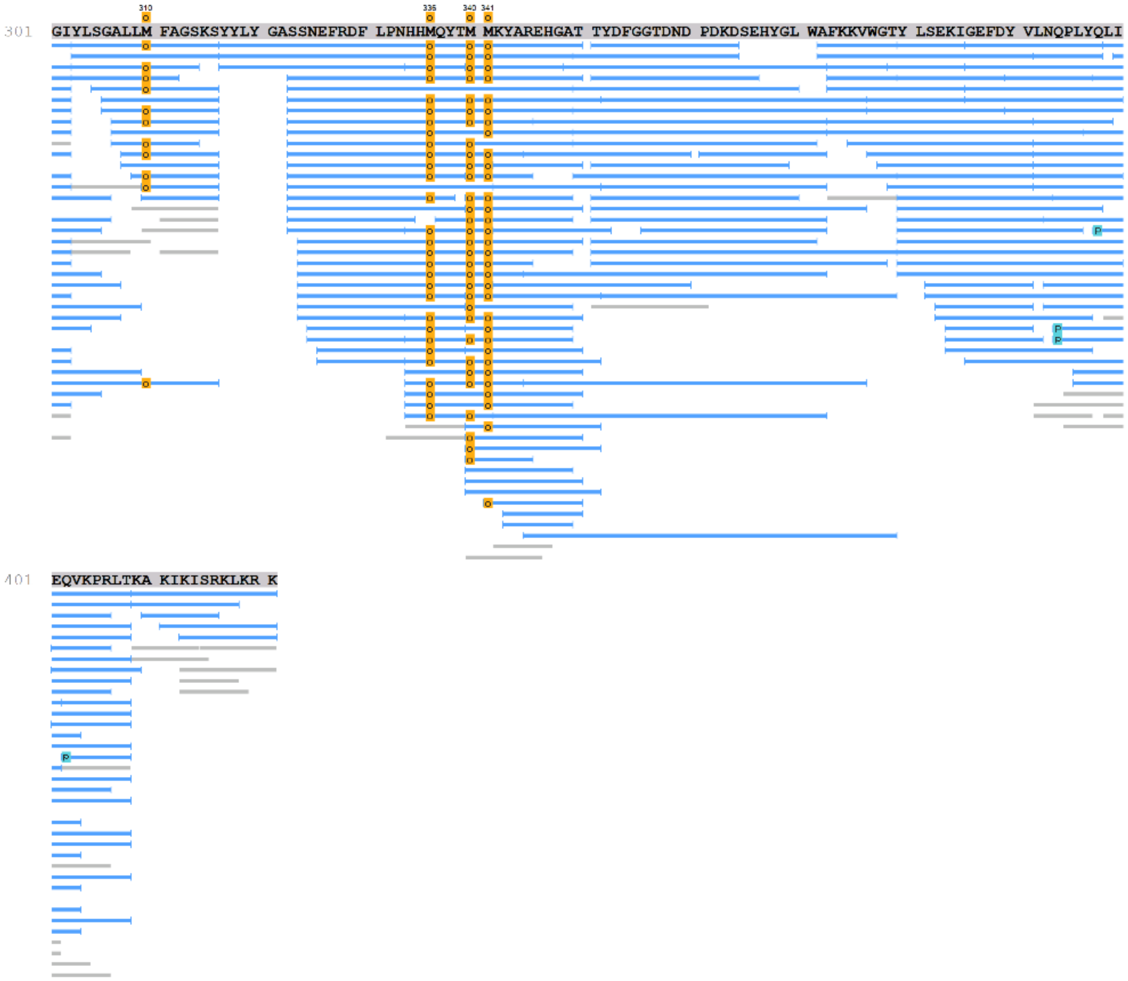

Figure S5c. Identification of *in vitro* phosphorylation site of FemX.

Recombinant FemX were incubated with 20 mM ATP and Stk_{KD} as well as Stp in kinase buffer for 1 h at 37 °C. After SDS-PAGE, the appropriate FemX protein band was digested with elastase and the peptides were measured by LC-MS/MS. Phosphorylated peptides are marked with red boxes.

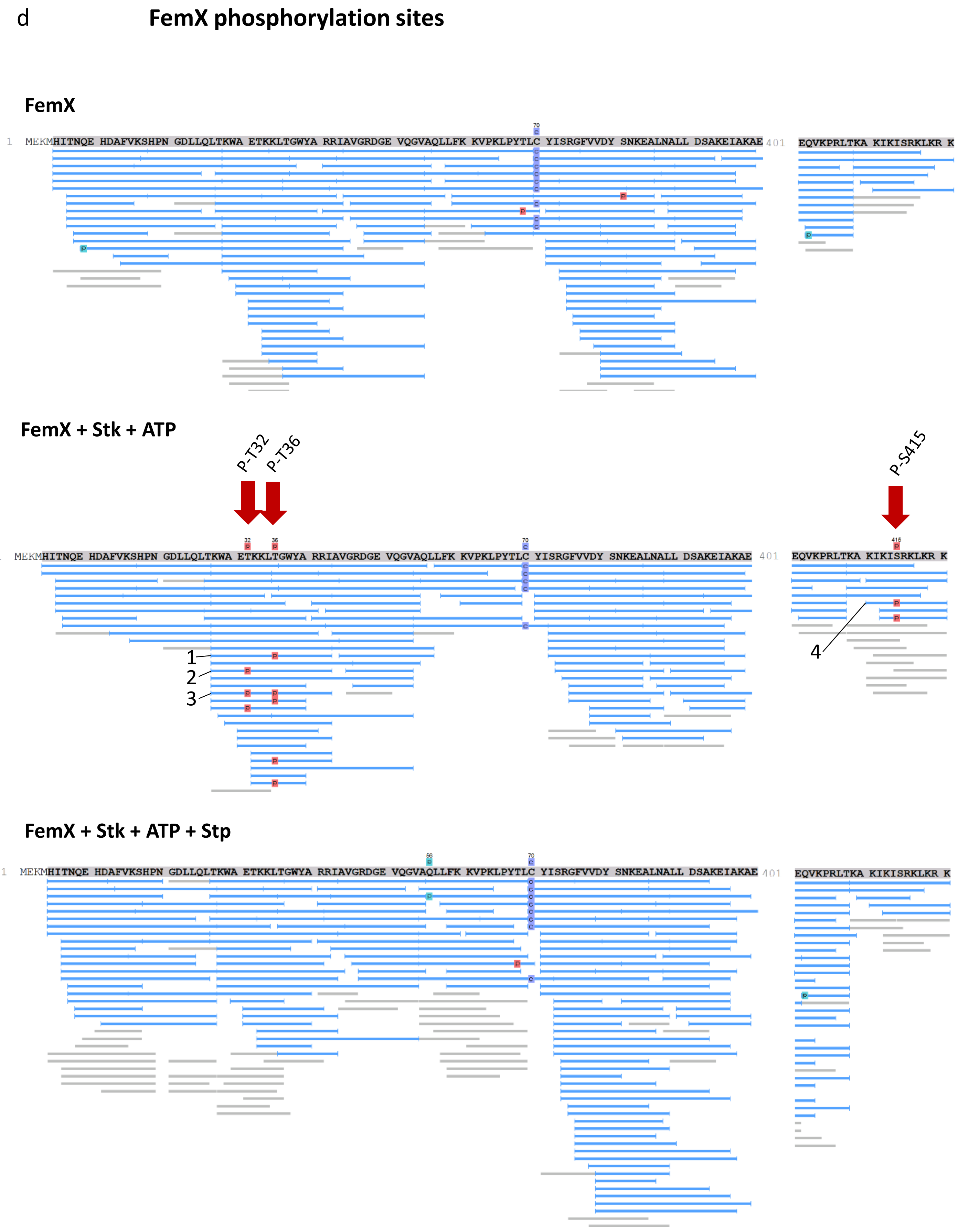

Figure S5d. Identification of *in vitro* phosphorylation site of FemX.
Stk_{KD} phosphorylated FemX. Mass spectrometry analysis determined the Stk-mediated phosphorylation sites of FemX at Thr32, Thr36 and Ser415. Stp was able to completely dephosphorylate these Stk-mediated phosphorylation sites.

e

FemX phosphorylation sites

peak 1_P-T32

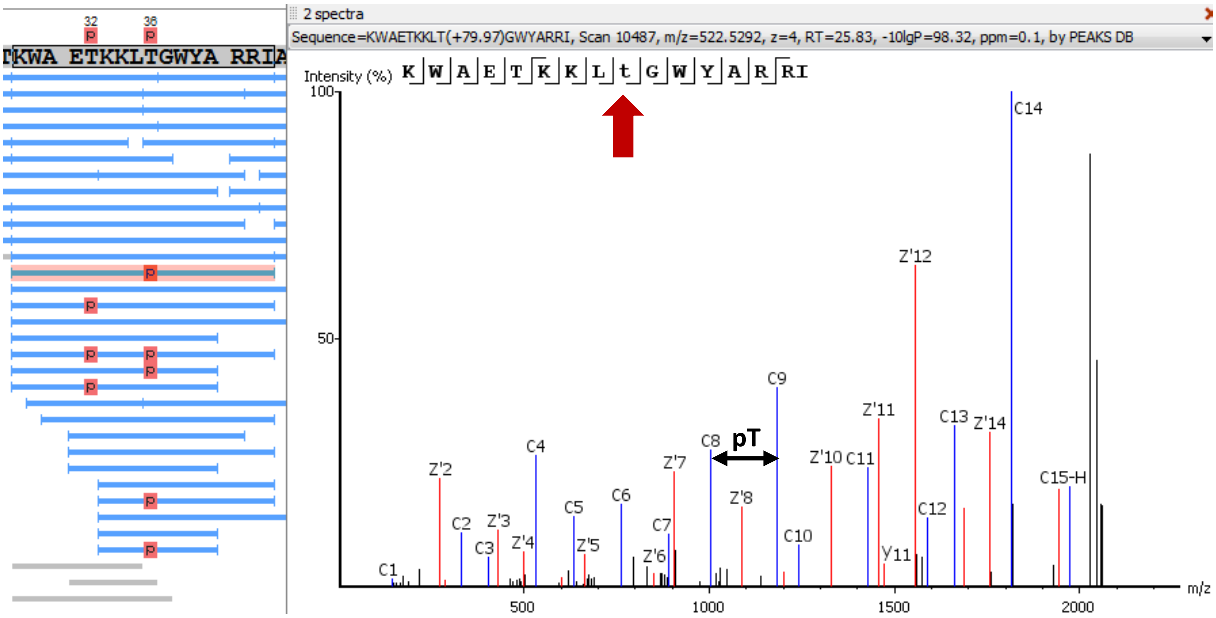

peak 2_P-T36

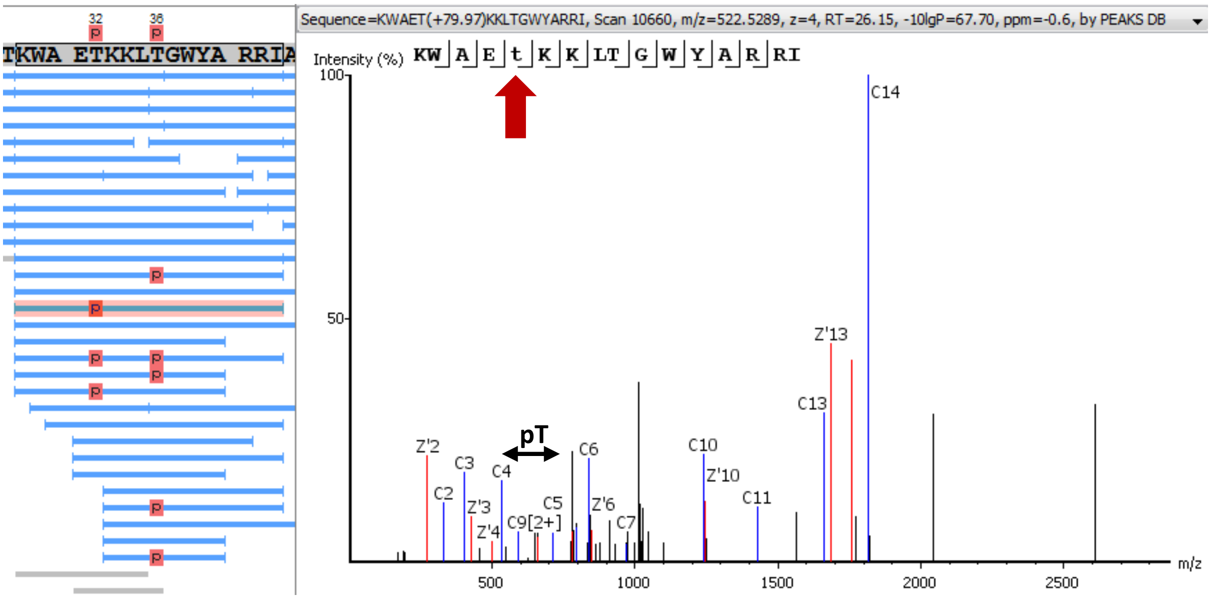

e

FemX phosphorylation sites

peak 3_P-T32_P-T36

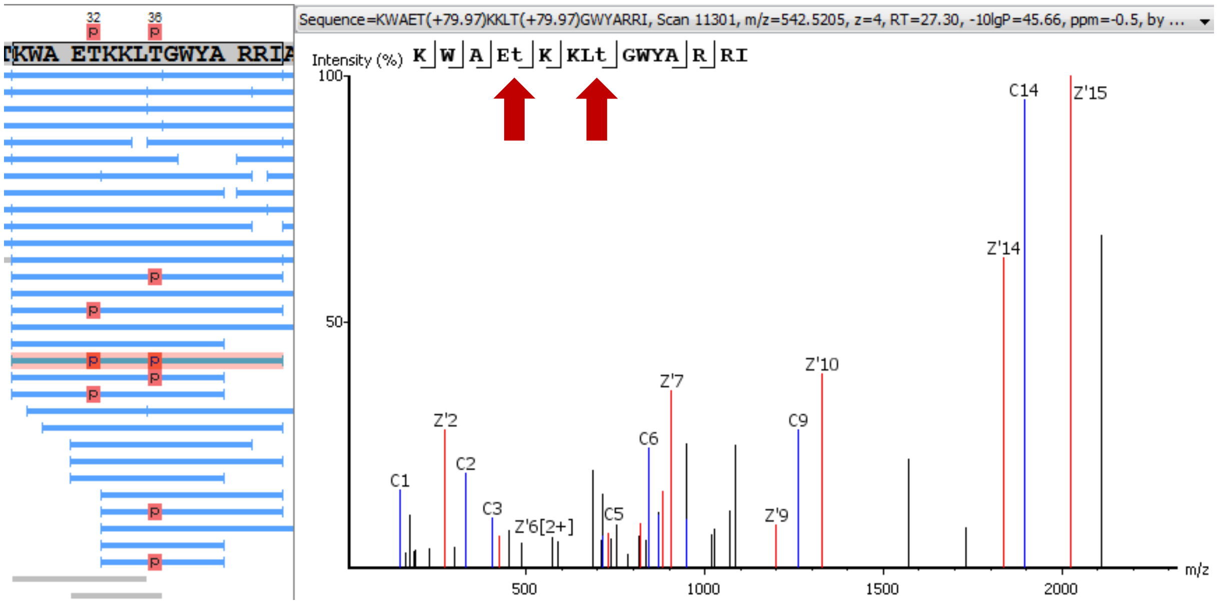

peak 4_P-S415

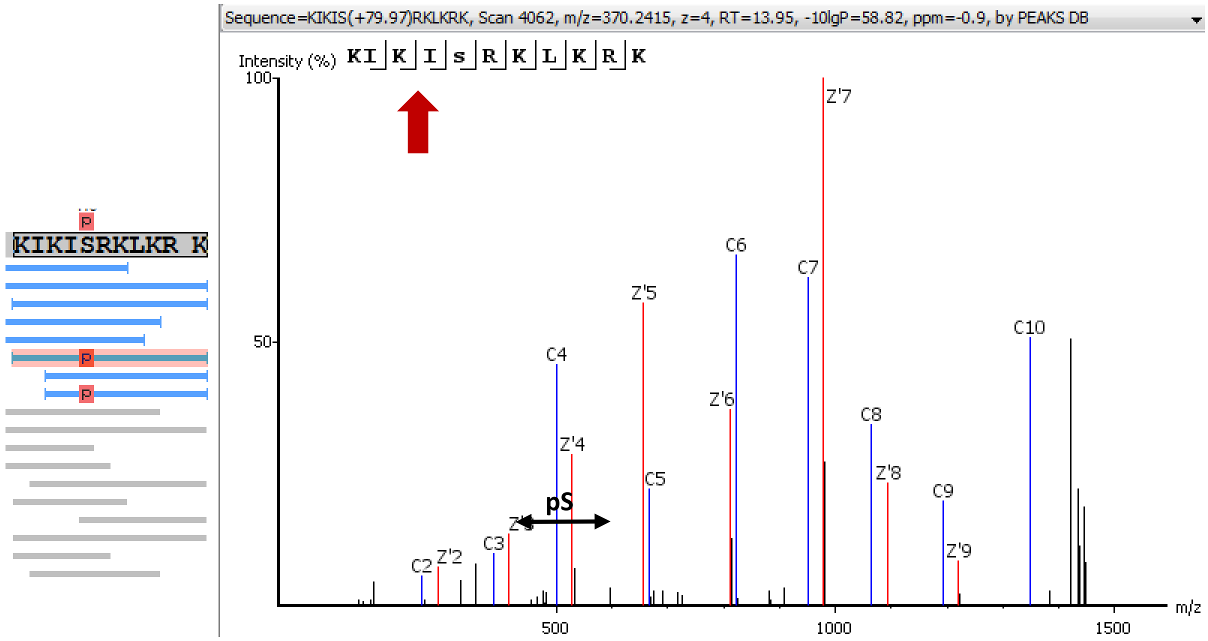

Figure S5e. MS/MS spectra of tryptic FemX peptides containing the Stk phosphorylation sites. Mass spectrometry analysis determined the Stk-mediated phosphorylation sites of FemX at Thr32, Thr36 and Ser415. We detected mono- (peak 1 and 2) and diphosphorylated (peak 3) phosphothreonine peptides of FemX.

f **FemA**, w/o ATP, w/o Stk, w/o Stp

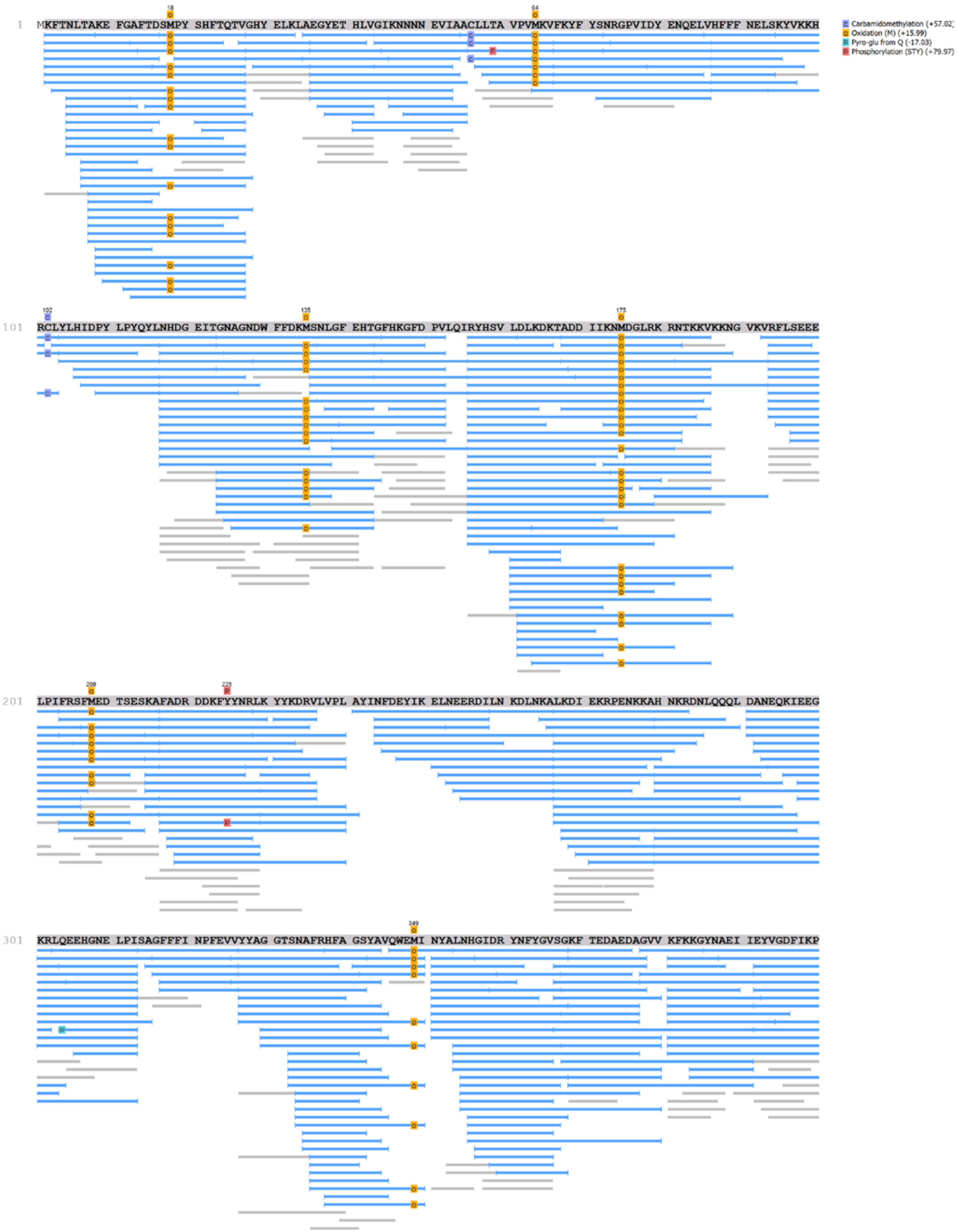

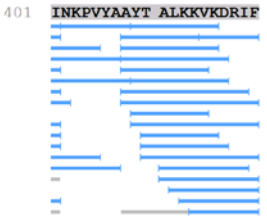

Figure S5f. Identification of *in vitro* phosphorylation site of FemA. Recombinant FemA were incubated without ATP and Stk_{KD} in kinase buffer for 1 h at 37 °C. After SDS-PAGE, the appropriate FemA protein band was digested with elastase and the peptides were measured by LC-MS/MS. Phosphorylated peptides are marked with red boxes.

g **FemA** + ATP + **Stk**, w/o Stp

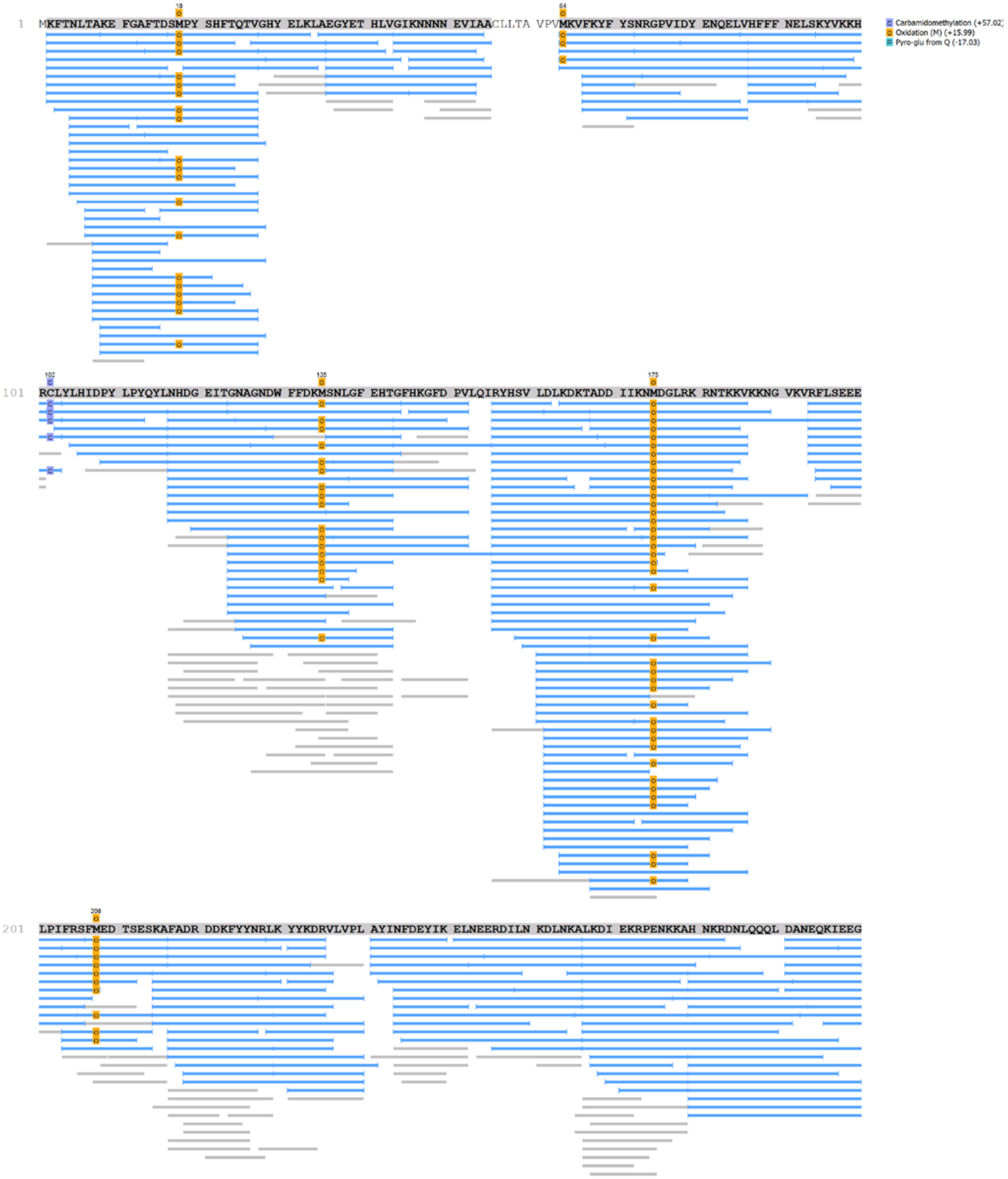

g **FemA** + ATP + **Stk**, w/o Stp

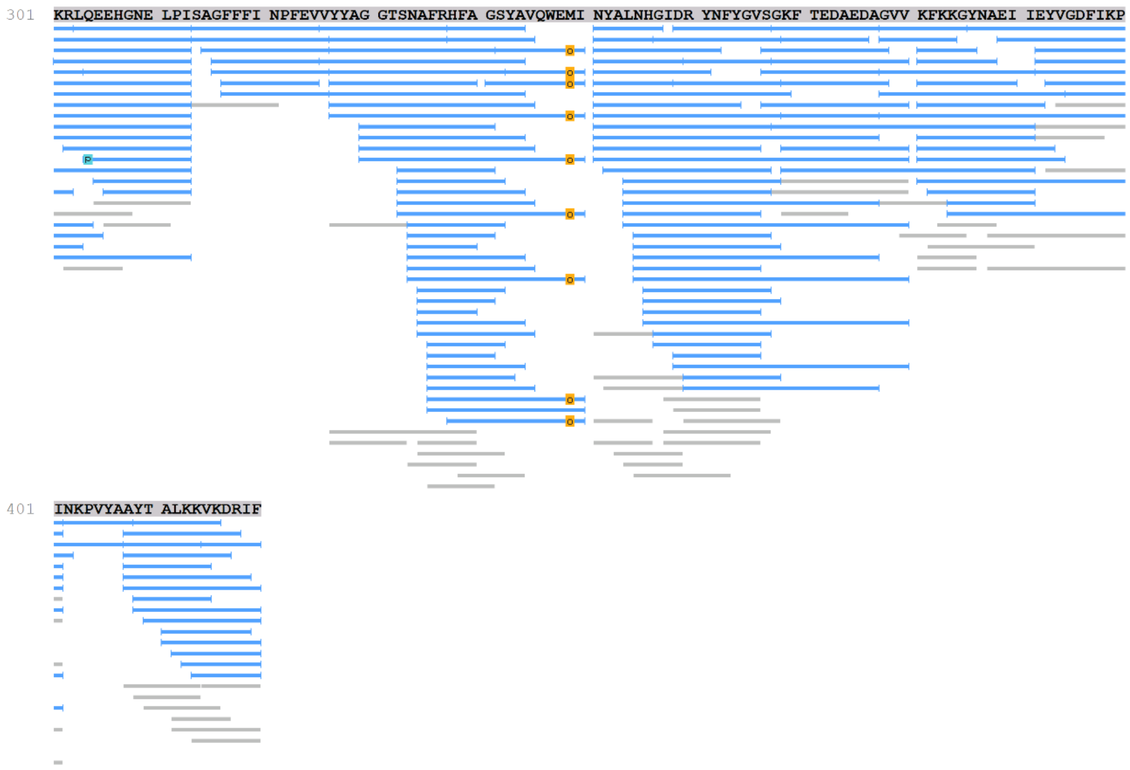

Figure S5g. Identification of *in vitro* phosphorylation site of FemA.
Recombinant FemA were incubated with 20mM ATP and Stk_{KD} in kinase buffer for 1 h at 37 °C. After SDS-PAGE, the appropriate FemA protein band was digested with elastase and the peptides were measured by LC-MS/MS. Phosphorylated peptides are marked with red boxes.

h **FemA** + ATP + **Stk** + **Stp**

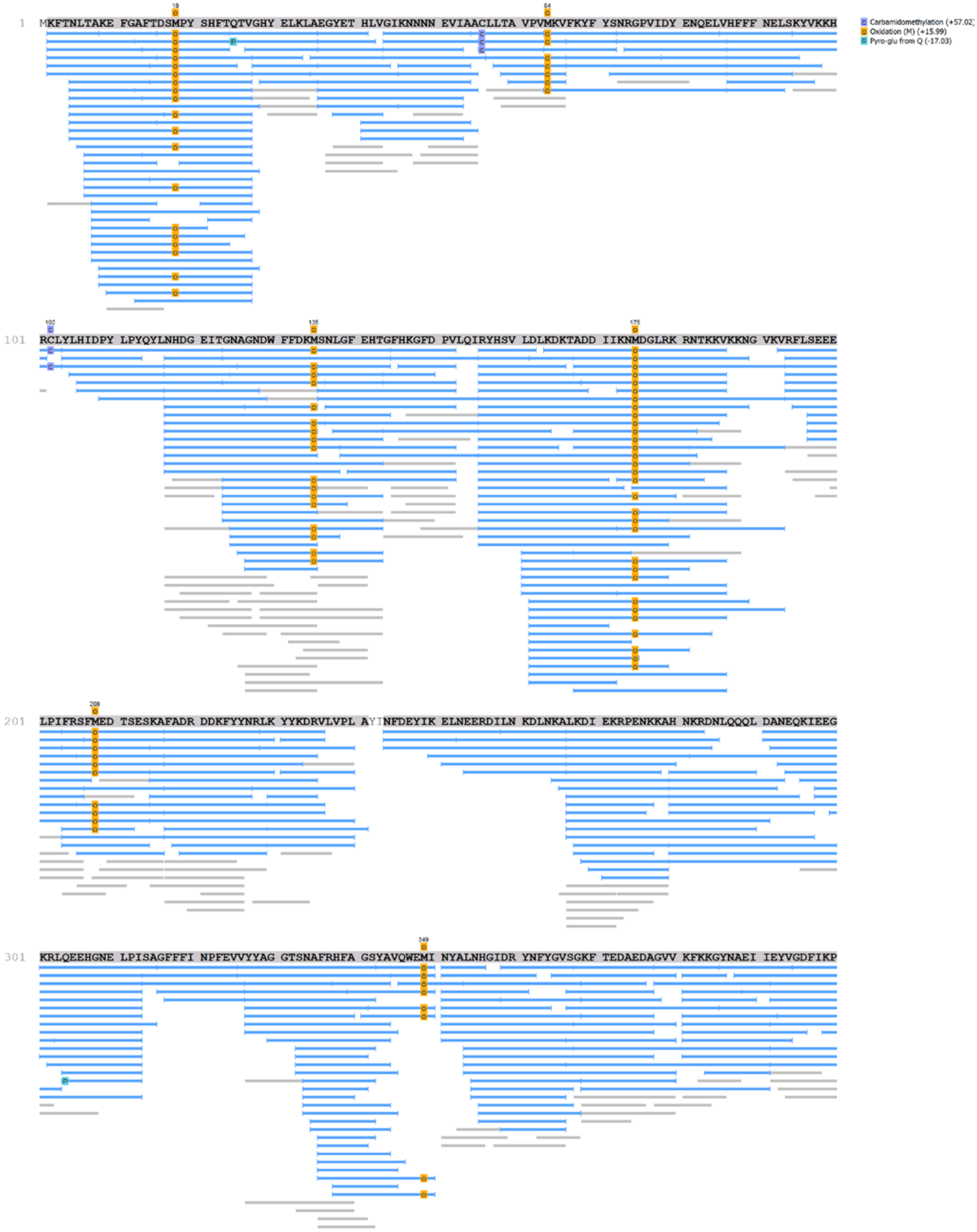

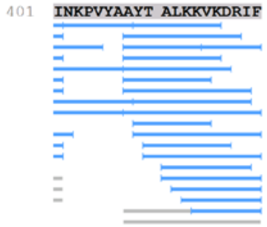

Figure S5h. Identification of *in vitro* phosphorylation site of FemA. Recombinant FemA were incubated with 20 mM ATP and Stk_{KD} as well as Stp in kinase buffer for 1 h at 37 °C. After SDS-PAGE, the appropriate FemA protein band was digested with elastase and the peptides were measured by LC-MS/MS. Phosphorylated peptides are marked with red boxes.

i

FemB, w/o ATP, w/o Stk, w/o Stp

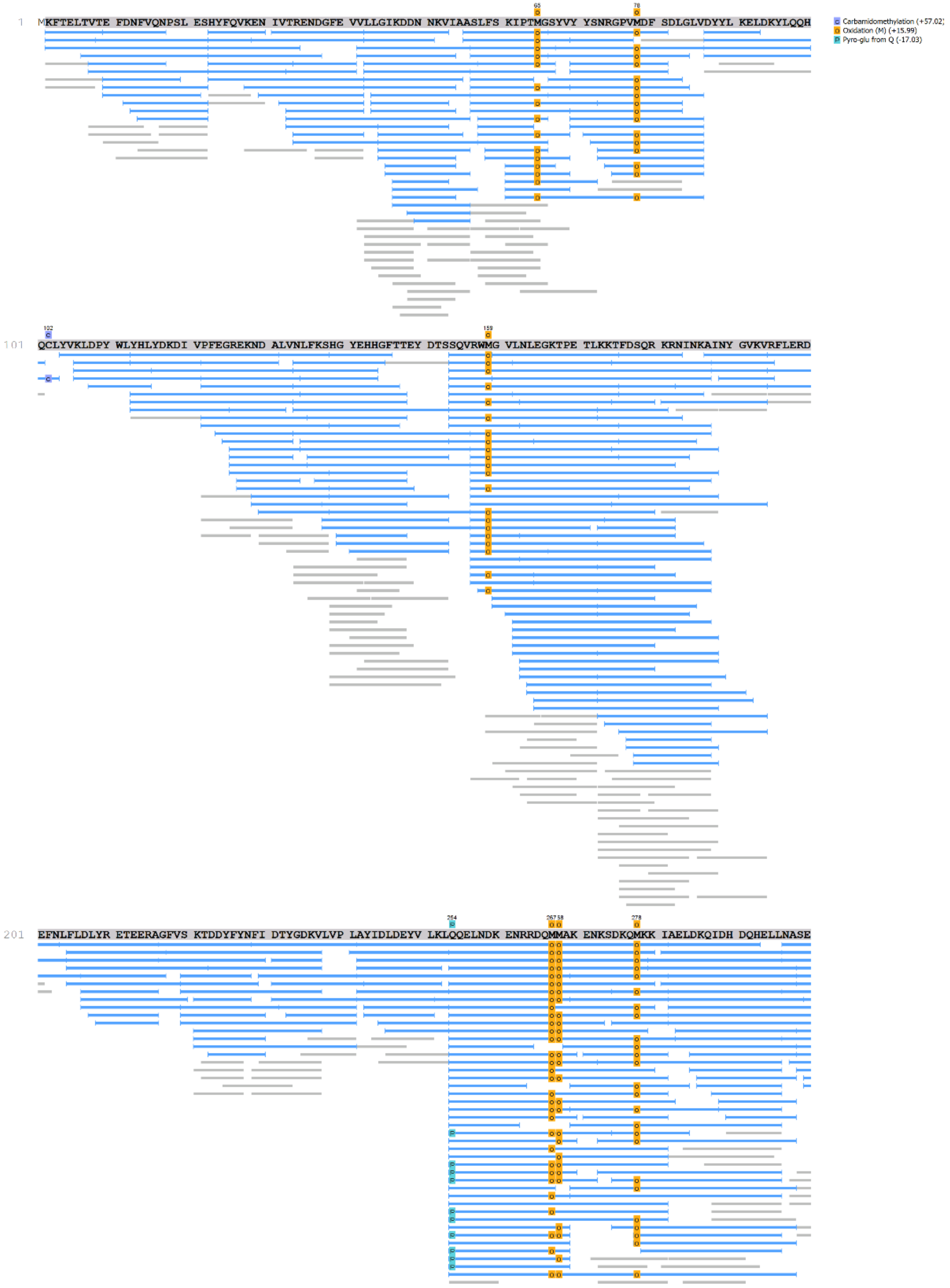

i **FemB,** w/o ATP, w/o Stk, w/o Stp

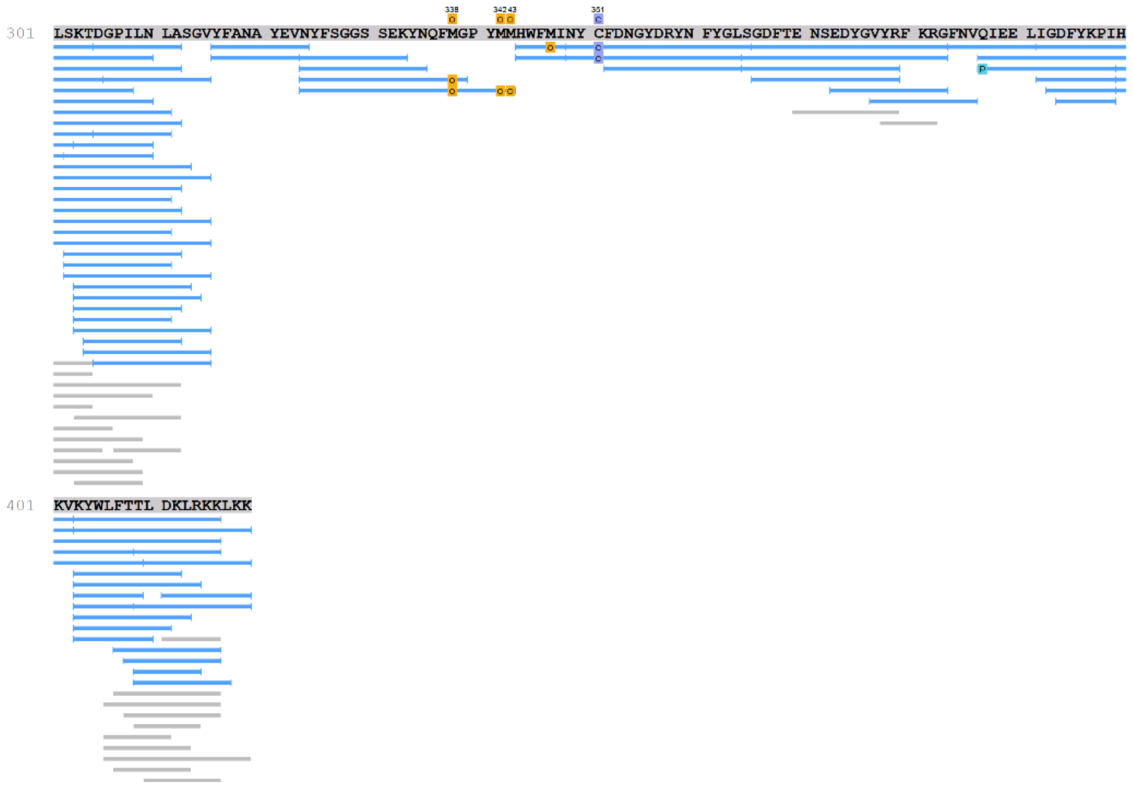

Figure S5i. Identification of *in vitro* phosphorylation site of FemB.
Recombinant FemB were incubated without ATP and Stk_{KD} in kinase buffer for 1 h at 37 °C. After SDS-PAGE, the appropriate FemB protein band was digested with elastase and the peptides were measured by LC-MS/MS. Phosphorylated peptides are marked with red boxes.

j **FemB +** ATP **+ Stk**, w/o Stp

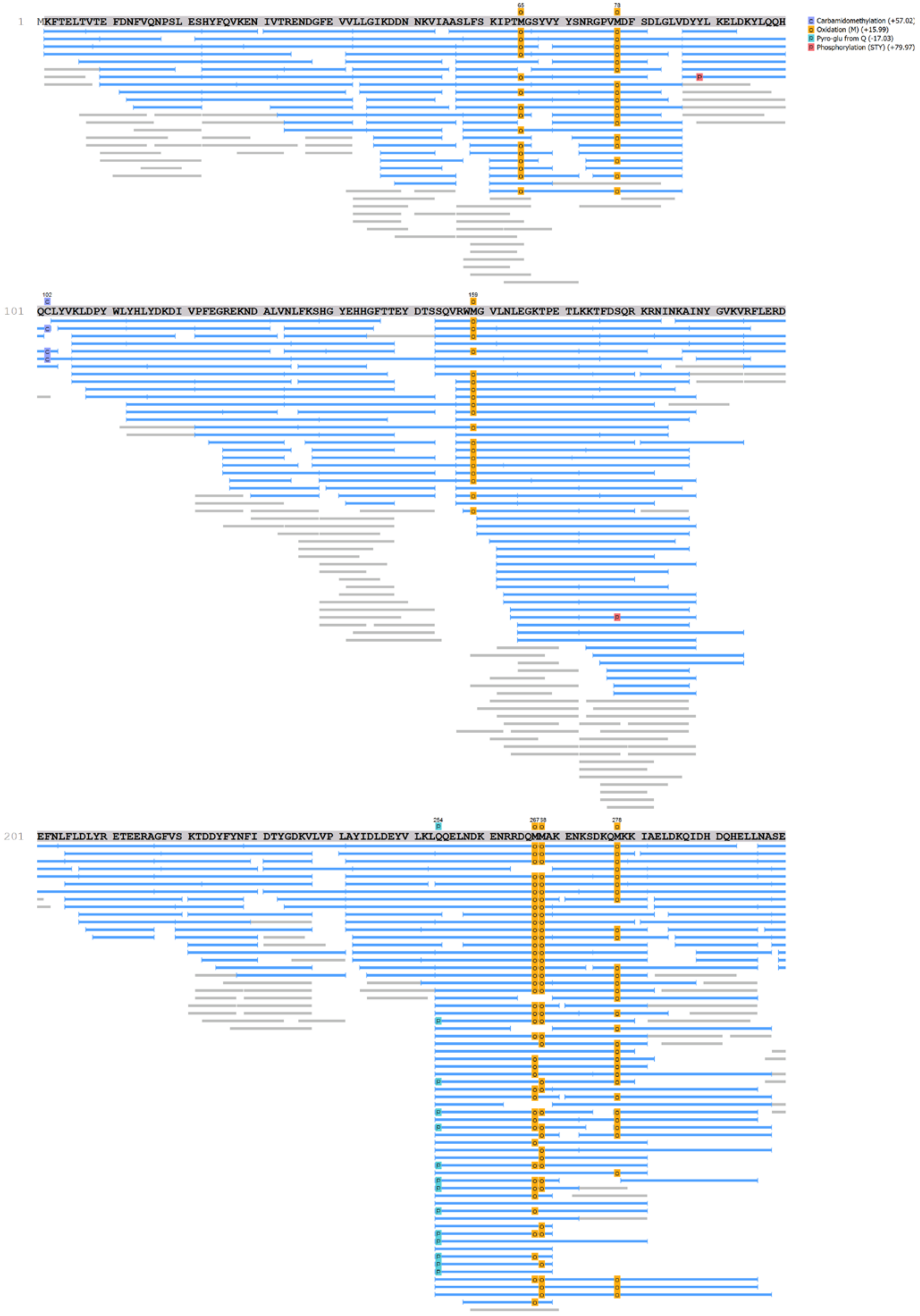

j **FemB + ATP + Stk**, w/o Stp

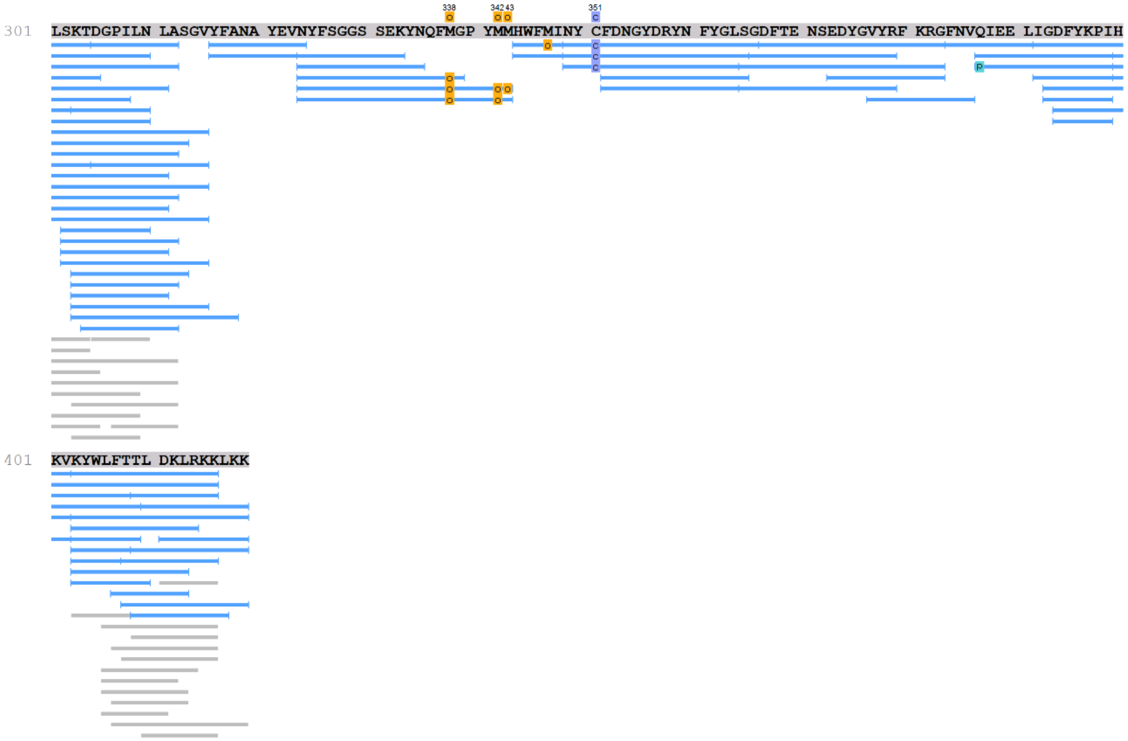

Figure S5j. Identification of *in vitro* phosphorylation site of FemB.
Recombinant FemB were incubated with 20 mM ATP and Stk_{KD} in kinase buffer for 1 h at 37 °C. After SDS-PAGE, the appropriate FemB protein band was digested with elastase and the peptides were measured by LC-MS/MS. Phosphorylated peptides are marked with red boxes.

k **FemB + ATP + Stk + Stp**

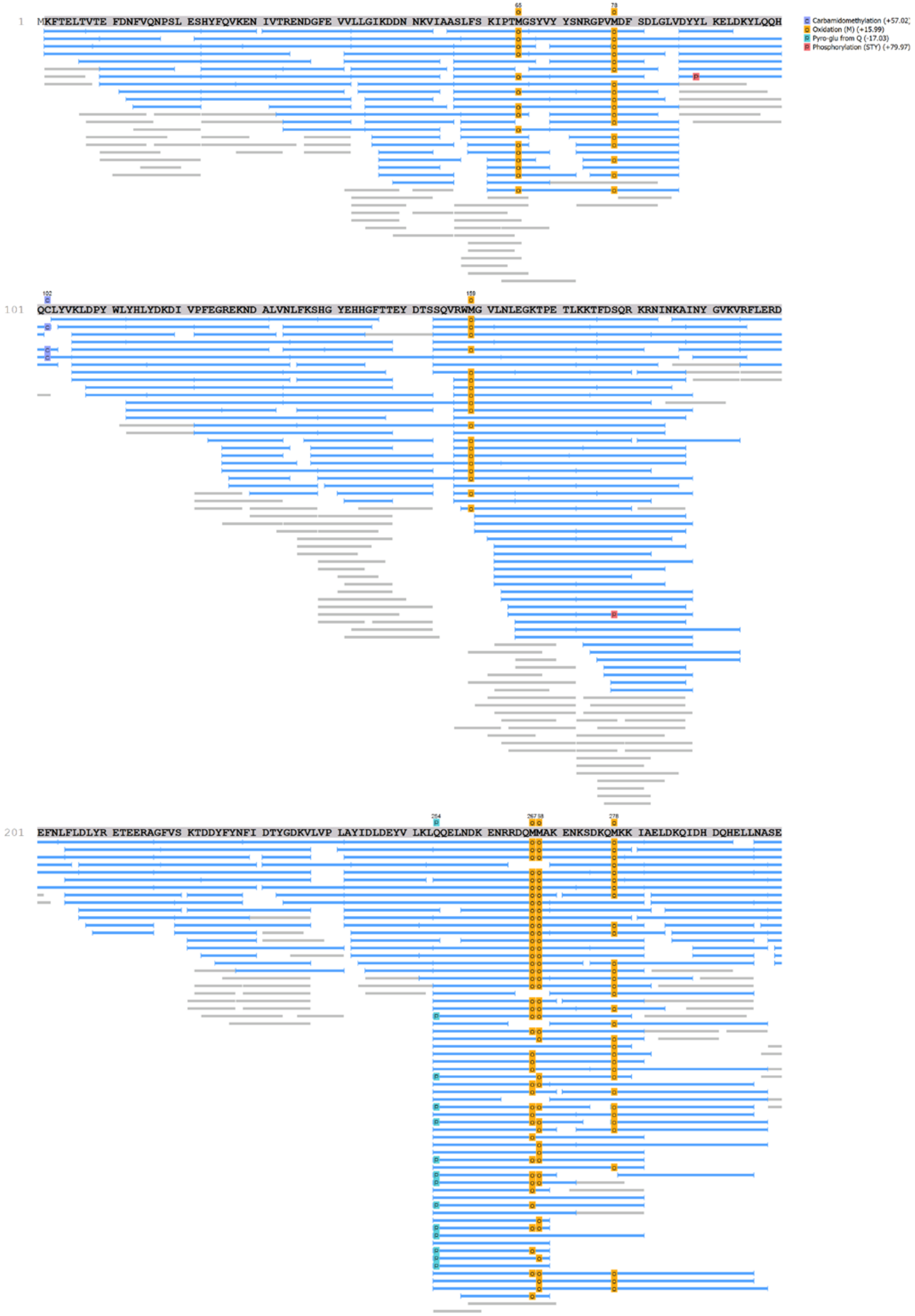

k **FemB + ATP + Stk + Stp**

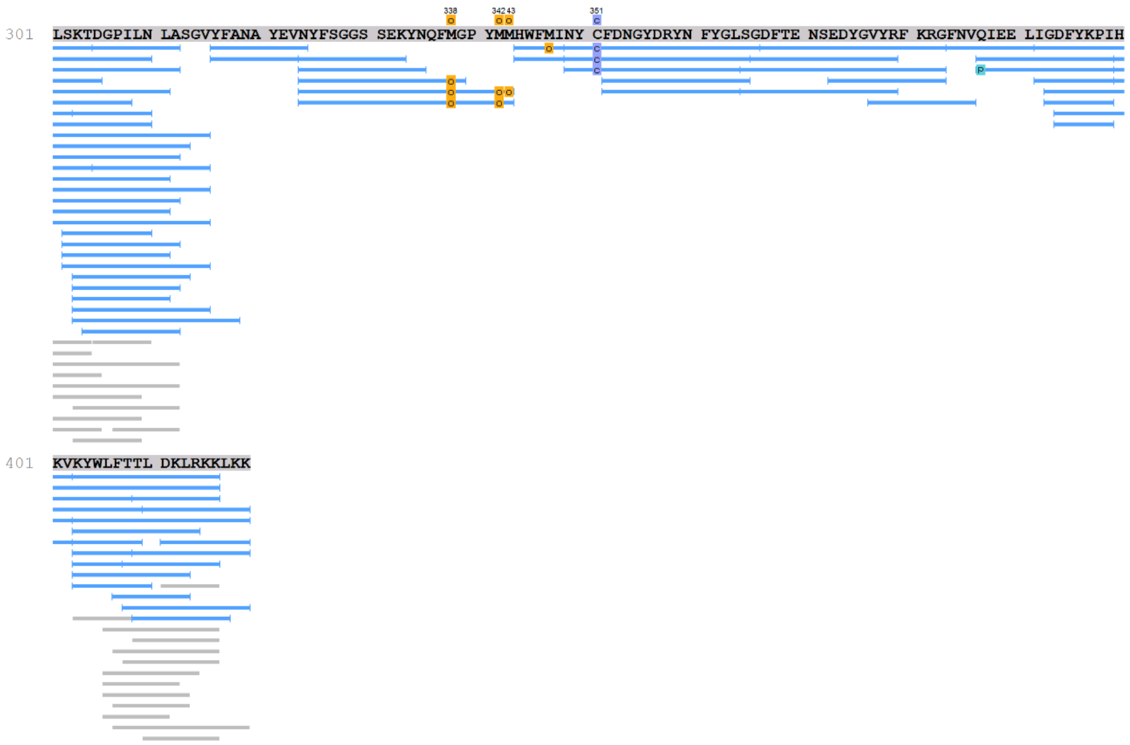

Figure S5k. Identification of *in vitro* phosphorylation site of FemB.
Recombinant FemB were incubated with 20 mM ATP and Stk_{KD} as well as Stp in kinase buffer for 1 h at 37 °C. After SDS-PAGE, the appropriate FemB protein band was digested with elastase and the peptides were measured by LC-MS/MS. Phosphorylated peptides are marked with red boxes.

Table S6. Minimal inhibitory concentration (MIC) of cell wall-active antibiotics for *S. aureus* NewmanHG wt and mutant strains.

	MICs [µg/ml] of *S. aureus*			
	wt	Δ*stk*	Δ*stp*	Δ*stk*Δ*stp*
fosfomycin	8	16	4	4
D-cycloserine	32	32	16	32
tunicamycin	32	4	16	4
ramoplanin	0.06	0.03	0.06	0.03
nisin	128	128	256	128
methicillin	2	1	2	1
penicillin G	0.25	0.13	0.25	0.13
vancomycin	2	2	2	2
daptomycin	2	2	2	2
bacitracin	32	32	32	32

Figure S7

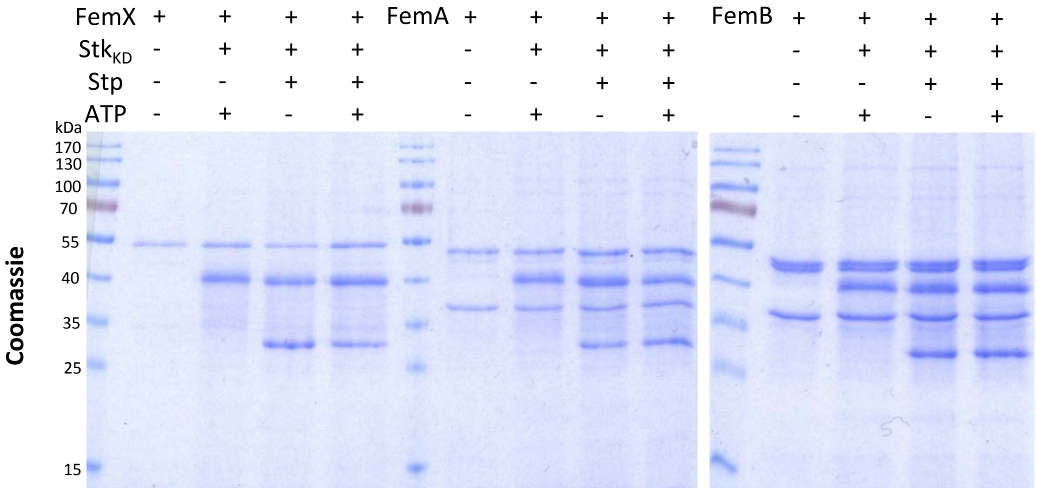

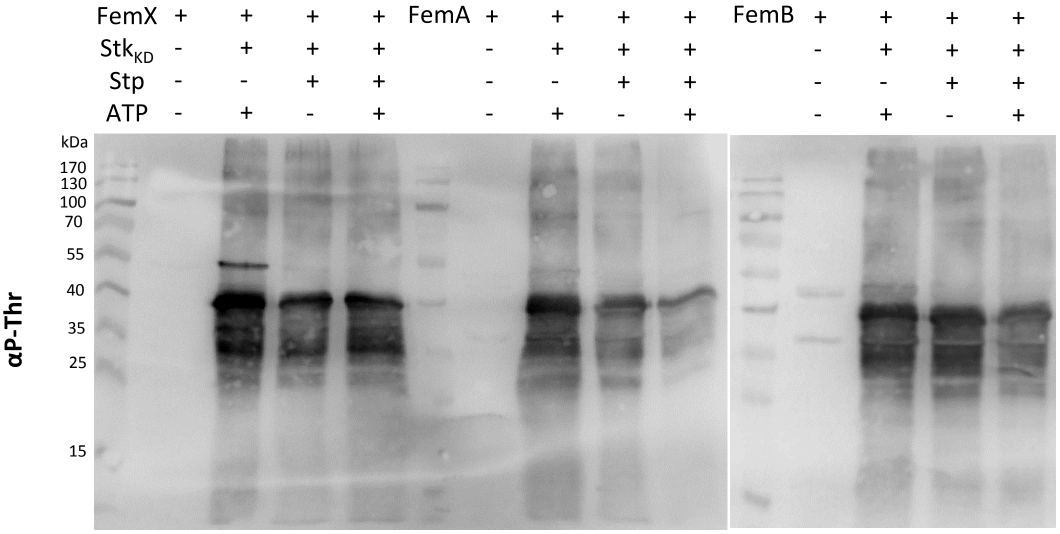

Figure S7. Stk/Stp *in vitro* kinase/phosphatase assay.
One µg recombinant protein (FemX, FemA or FemB) was incubated with 1 µg Stk kinase domain (Stk_{KD}) in kinase buffer with or without 20 mM ATP for 1 h at 37 °C. For the dephosphorlyation reaction, 1 µg Stp was added to the reaction mixture. Each reaction was stopped by addition of 5 x SDS sample buffer and heating for 5 min at 95 °C. The phosphorylated proteins were detected by Western blot using an anti-phosphothreonine antibody (αP-Thr #9381S, Cell Signaling, Germany) and were visualized using the ImageQuant LAS 4000 imaging system (GE Healtcare). The upper panel shows the loading control with Coomassie-blue staining; the bottom panel shows phosphorylated proteins detected by αP-Thr Western blot. M: Marker PageRuler™ Prestained Protein Ladder (Thermo Fisher Scientific Inc, USA)

Table S8. Bacterial strains used in this study

strains	characteristic	source or reference
S. aureus NewmanHG	*S. aureus* Newman, *saeS*$^{+}$	Maniero et al.[1]
S. aureus NewmanHG *Δstk*	*stk* deletion, *stk*::*ermB*, Erm^{R}	This work
S. aureus NewmanHG *Δstp*	*stp* deletion, *stp*::*ermB*, Erm^{R}	This work
S. aureus NewmanHG *ΔstkΔstp*	*stk*/stp deletion, *stk/stp*::*ermB*, Erm^{R}	This work
S. aureus RN4220	NCTC 8325-4-r; 11 bp Deletion in *rsbU*	Novick, R.
E. coli DH5α	F^{-} *endA1 hsdR17 supE44 thi-1 recA1 gyrA96 relA1*(*A*) Δ(*argF-lac*)*U169* Φ80d*lacZ*ΔM15	MBI-Fermentas
E.coli DC10B	mcrA Δ(mrr-hsdRMS-mcrBC) ϕ80lacZΔM15 ΔlacX74 recA1 araD139 Δ(ara-leu)7697 galU galK rpsL endA1 nupG Δdcm	Monk, et al. [2]
E.coli BL21(DE3)	F^{-}*ompT hsdS*B (rS_ mS_) *gal dcm* (DE3)	Novagen
E. coli BTH101	F-, *cya-99*, *araD139, galE15, galK16, rpsL1 (Str r)*, *hsdR2, mcrA1, mcrB1*	Euromedex

Table S9. *E. coli* bacterial two-hybrid strains used in this study

strains	characteristic	source or reference
B04	*E.coli* DH5α p25N (pKNT25), Kan^R	Euromedex
B05	*E.coli* DH5α p25C (pKT25) Kan^R	Euromedex
B06	*E.coli* DH5α p18C (pUT18C), Amp^R	Euromedex
B07	*E.coli* DH5α p18N (pUT18), Amp^R	Euromedex
B08	*E.coli* DH5α pUT18C-zip, Amp^R	Euromedex
B09	*E.coli* DH5α pKT25-zip, Kan^R	Euromedex
B15	*E.coli* DH5α p25C-Stk (NWMN_1130)	This work
B17	*E.coli* DH5α p18C-Stk (NWMN_1130)	This work
B18	*E.coli* DH5α p25N-Stp (NWMN_1129)	This work
B19	*E.coli* DH5α p25C-Stp (NWMN_1129)	This work
B20	*E.coli* DH5α p18N-Stp (NWMN_1129)	This work
B21	*E.coli* DH5α p18C-Stp (NWMN_1129)	This work
B37	*E.coli* DH5α p18C-MurC (NWMN_1633)	This work
B63	*E.coli* DH5α p18C-DivIB (pGL544)	Steele et al, 2011[3]
B64	*E.coli* DH5α p18C-FtsA (pGL545)	Steele et al, 2011[3]
B65	*E.coli* DH5α p18C-FtsL (pGL546)	Steele et al, 2011[3]
B66	*E.coli* DH5α p18C-Pbp2 (pGL547)	Steele et al, 2011[3]
B67	*E.coli* DH5α p18C-FtsZ (pGL562)	Steele et al, 2011[3]
B68	*E.coli* DH5α p18C-DivIC (pGL554)	Steele et al, 2011[3]
B69	*E.coli* DH5α p18C-GpsB (pGL570)	Steele et al, 2011[3]
B70	*E.coli* DH5α p18C-SepF (pGL572)	Steele et al, 2011[3]
B71	*E.coli* DH5α p18C-FtsW (pALB6)	Steele et al, 2011[3]
B72	*E.coli* DH5α p18C-RodA (pALB14)	Steele et al, 2011[3]
B73	*E.coli* DH5α p18C-EzrA (pVF32)	Steele et al, 2011[3]
B92	*E.coli* DH5α p18C-Pbp2	D. Lopez
B93	*E.coli* DH5α p18C-Pbp3	D. Lopez
B117	*E.coli* DH5α p18C-MurA (NWMN_2004)	This work
B119	*E.coli* DH5α p18C-MurB (NWMN_0707)	This work
B121	*E.coli* DH5α p18C-MurD (NWMN_1093)	This work
B123	*E.coli* DH5α p18C-MurE (NWMN_0888)	This work
B125	*E.coli* DH5α p18C-MurF (NWMN_1986)	This work
B127	*E.coli* DH5α p18C-MraY (NWMN_1092)	This work
B129	*E.coli* DH5α p18C-MurG (NWMN_1330)	This work

Table S9. *E. coli* bacterial two-hybrid strains used in this study

strains	characteristic	source or reference
B131	*E.coli* DH5α p18C-GlmS (NWMN_2056)	This work
B133	*E.coli* DH5α p18C-GlmM (NWMN_2062)	This work
B135	*E.coli* DC10B p18C-GlmU (NWMN_0462, *gcaD*)	This work
B173	*E.coli* DC10B p18C-FemX (NWMN_2163, *fmhB*)	This work
B175	*E.coli* DC10B p18C-FemA (NWMN_1286)	This work
B177	*E.coli* DC10B p18C-FemB (NWMN_1287)	This work
B203	*E.coli* DC10B p18C-Pbp4 (NWMN_0612)	This work
B207	*E.coli* DC10B p18C-Ddl (NWMN_1987)	This work
B209	*E.coli* DC10B p18C-OatA (NWMN_2467)	This work
B213	*E.coli* DC10B p18C-MurI (NWMN_1063)	This work
B215	*E.coli* DC10B p18C-MurT (NWMN_1830)	This work
B217	*E.coli* DC10B p18C-Sle1 (NWMN_0429)	This work
B219	*E.coli* DC10B p18C-LytH (NWMN_1534)	This work
B221	*E.coli* DC10B p18C-SsaA (NWMN_2443)	This work
B223	*E.coli* DC10B p18C-Mgt (NWMN_ 1766)	This work
B225	*E.coli* DC10B p18C-GatD (NWMN_1829)	This work
B226	*E.coli* DC10B p25C-FemX (NWMN_2163, *fmhB*)	This work
B227	*E.coli* DC10B p25C-FemA (NWMN_1286)	This work
B228	*E.coli* DC10B p25C-FemB (NWMN_1287)	This work
B233	*E.coli* DC10B p25C-MurG (NWMN_1330)	This work

Table S10. Plasmids used in this study

plasmid	characteristic	source or reference
pGEM-T	Amp^r	Promega
pEC1	pUC18 *ermB*, Amp^r, Em^r	Brückner, 1992
pBT2	shuttle vector, Amp^r, Cm^r	Brückner, 1997
pET-28a(+)	His_6 expression vector, Kan^r	Novagen
pET-28a-Stk_{KD}	His_6-$Stk_{1\text{-}291}$, cloned in pET28a(+), Kan^r	This study
pET-28a-Stp	His_6-Stp, cloned in pET28a(+), Kan^r	This study
pET-28a-FemX	His_6-FemX, cloned in pET28a(+), Kan^r	This study
pET-28a-FemA	His_6-FemA, cloned in pET28a(+), Kan^r	This study
pET-28a-FemB	His_6-FemB, cloned in pET28a(+), Kan^r	This study

Table S11. Primer used in this study

Primer	sequence	restriction enzyme
ermBEcoRI	CGGAATTCGGTGACATCTCTCTATTGTG	EcoRI
ermBPstI	AACTGCAGGGAAGCTGTCAGTAGTATACC	PstI
stp-up_EcoRI	CGGAATTCTGGCTCGTTGAACAAGGTC	EcoRI
stp-up-BamHI	CGGGATCCACAAGCTGCGTCAATATCC	BamHI
stp-down-PstI	TACCTGCAGTACTCGCGGCTATTGAAGGTG	PstI
stp-down-EcoRV	CGGATATCAAGGGTGGTTCACCAACAAGC	EcoRV
stp1	CAACATGATGCGACAAAACC	
stp2	CTTGCGTCGTTTCACGTTTA	
pET_Stk_{KD}_fw	ATATTGGATCCataggtaaaataataaatgaacg	BamHI
pET_ Stk_{KD} _rev	ATATTCTCGAGTTAtaccgctatcgttttc	EcoRI
pET_Stp_fw	*CATATG*CTAGAGGCACAATTTTTTACTG	NdeI
pET_Stp_rev	CGC*CCTAGG*TCATACTTTATCACCTTCAATAGGCCG	BamHI
pET FemX_fw	ATATGAATTCgaaaagatgcatatcactaatca	EcoRI
pET FemX_rev	TATACTCGAGctattttcgttttaatttacgagatat	XhoI
pET FemA_fw	ATATGGATCCaagtttacaaatttaacagctaaagag	BamHI
pET FemA_rev	TATACTCGAGctaaaaaattctgtctttaactttttttaa	XhoI
pET FemB_fw	ATATGGATCCaaatttacagagttaactgttaccg	BamHI
pET FemB_rev	TATACTCGAGctatttctttaattttttacgtaatttatc	XhoI

Table S12. Primer for constructing bacterial two-hybrid vectors

primer	sequence	restriction enzyme
B007 C-Stk (s)	ATTGGATCCGATAGGTAAAATAATAAATGAACGATATAAAAtTG	BamHI
B008 C-Stk (a)	TAAGAATTCTTATACATCATCATAGCTGACTTCTTTTTC	EcoRI
B012 C-Stp (s)	ATTGGATCCGCTAGAGGCACAATTTTTTACTGATACTGGA	BamHI
B013 C-Stp (a)	TAAGAATTCTCATACTTTATCACCTTCAATAGCCGC	EcoRI
B020 C-MurC (s)	ATTGGATCCGACACACTATCATTTTGTCGGAAT	BamHI
B021 C-MurC (a)	TAAGAATTCCTAAAACGCATTTTTCATGCCTAATTTA	EcoRI
B026 N-Stk (s)	ATTGGATCCATGATAGGTAAAATAATAAATGAACGATATAAAATG	BamHI
B027 N-Stk (a)	TAAGAATTCGCTACATCATCATAGCTGACTTCTTTTTC	EcoRI
B031 N-Stp (s)	ATTGGATCCGATGCTAGAGGCACAATTTTTTACTGATACTGGA	BamHI
B032 N-Stp (a)	TAAGAATTCGCTACTTTATCACCTTCAATAGCCGC	EcoRI
B045 p25C (s)	CGGCGGATATCGACAT	sequencing
B046 p25C (a)	CTGGCGAAAGGGGGAT	sequencing
B047 p25/18N (s)	CACTTTATGCTTCCGGCTC	sequencing
B048 p25N (a)	CTTGATGCCATCGAGTACG	sequencing
B049 p18C (s)	GAGCGGACGTTCGAAGTT	sequencing
B050 p18C (a)	GTTGGCGGGTGTCGG	sequencing
B051 p18N (a)	TCCACAACAAGTCGATGC	sequencing
B045 p25C (s)	CGGCGGATATCGACAT	sequencing
B046 p25C (a)	CTGGCGAAAGGGGGAT	sequencing
B070 C-MurA_fw	ATATTGGATCCGgataaaatagtaatcaaaggtggaa	BamHI
B071 C-MurA_rev	TATTAGAATTCttaatcgttaatacgttcaatgtc	EcoRI
B074 C-MurB_fw	ATATTGGATCCGataaataaagacatctatcaagctttac	BamHI
B075 C-MurB_rev	TATAAGAATTCttacgattcctttggatgtt	EcoRI
B078 C-MurD_fw	ATATTGGATCCGcttaattatacagggttagaaaataaaaatg	BamHI
B079 C-MurD_rev	TATAAGAATTCttaataagatggtaaatgggca	EcoRI
B082 C-MurE_fw	ATATTGGATCCGgatgcaagtacgttgtttaaga	BamHI
B083 C-MurE_rev	TATAAGAATTCttaatcaacagggccacc	EcoRI
B086 C-MurF_fw	ATATTGGATCCGattaatgttacattaaagcaaattcaa	BamHI
B087 C-MurF_rev	TATAAGAATTCctatgaaattaaagcatttaccact	EcoRI
B090 C-MraY_fw	ATATTGGATCCGatttttgtatatgcgttattagcg	BamHI
B091 C-MraY_rev	TATAAGAATTCttaatgcactccaatccataa	EcoRI
B094 C-MurG_fw	ATATTGGATCCGacgaaaatcgcatttacc	BamHI
B095 C-MurG_rev	TATAAGAATTCttaattcaatgcgtctttaatcat	EcoRI

Table S12. Primer for constructing bacterial two-hybrid vectors

primer	sequence	restriction enzyme
B098 C-GlmS_fw	ATATTGGATCCGtgtggaattgttggttatattg	BamHI
B099 C-GlmS_ rev	TATAAGAATTCttattccacagtaactgatttagc	EcoRI
B111 C-GlmM_fw	ATATTGGATCCGggaaaatattttggtacagacg	BamHI
B112 C-GlmM_rev	TATAAGAATTCttatttatctaatcccattttatcttga	EcoRI
B115 C-GlmU_fw	ATATTGGATCCGttcatgcgaagacacg	BamHI
B116 C-GlmU_rev	TATAAGAATTCttatttcctatatccttcttttgttg	EcoRI
B119 C-FemX_fw	TATAATCTAGAGgaaaagatgcatatcactaatc	XbaI
B120 C-FemX_rev	TATAAGAATTCctattttcgttttaatttacga	EcoRI
B123 C-FemA_fw	ATATTGGATCCGaagtttacaaatttaacag	BamHI
B124 C-FemA_rev	TATATGAATTCctaaaaaattctgtctttaactt	EcoRI
B127 C-FemB_fw	ATATTGGATCCGaaatttacagagttaactgt	BamHI
B128 C-FemB_rev	TATAAGAATTCctatttctttaatttttttacgtaat	EcoRI
B175 C-Pbp4_fw	ATTGGATCCGaaaaatttaatatctattatcatcatt	BamHI
B176 C-Pbp4_ rev	TAAGAATTCttattttctttttctaaataaacg	EcoRI
B183 C-Ddl_fw	ATTGGATCCGacaaaagaaaatatttgtatcg	BamHI
B184 C-Ddl_rev	ATTGAATTCttagtcaattttgtatttatttttc	EcoRI
B187 C-OatA_fw	ATTGGATCCGgatacaaaagactttaaacgtt	BamHI
B188 C-OatA_rev	ATTGAATTCttatttcttatttgtagcatgt	EcoRI
B194 C-MurI_fw	ATTGGATCCGaataaaccaataggtgtaatagac	EcoRI
B195 C-MurI_rev	ATTGAATTCctagtcattcactgatatacgtt	BamHI
B198 C-MurT_fw	ATTGGATCCGagacagtggacggca	EcoRI
B199 C-MurT_rev	TAAGAATTCttatgattgacctccttca	BamHI
B202 C Sle1_fw	ATTGGATCCGcaaaaaaaagtaattgcag	EcoRI
B203 C-Sle1_rev	TAAGAATTCttagtgaatatatctataattatttacttg	BamHI
B206 C-LytH_fw	ATTGGATCCGaaaaaaatagaggcatgg	EcoRI
B207 C-LytH_rev	TAAGAATTCctacgcagaaaaataaattt	BamHI
B210 C-SsaA_fw	ATTGGATCCGgaatataaaaagatactaattcgtt	EcoRI
B211 C-SsaA_rev	TAAGAATTCttagtaaatatagttaaagttacgtg	BamHI
B214 C-Mgt_fw	ATTGGATCCGaaaagaagcgataggtact	EcoRI
B215 C-Mgt_rev	TAAGAATTCttaacgatttaattgtgacata	BamHI
B218 C-GatD_fw	ATTGGATCCGcatgaattgactatttatcatt	EcoRI
B219 C-GatD_rev	TAAGAATTCttaacgagatttcttctgtc	BamHI

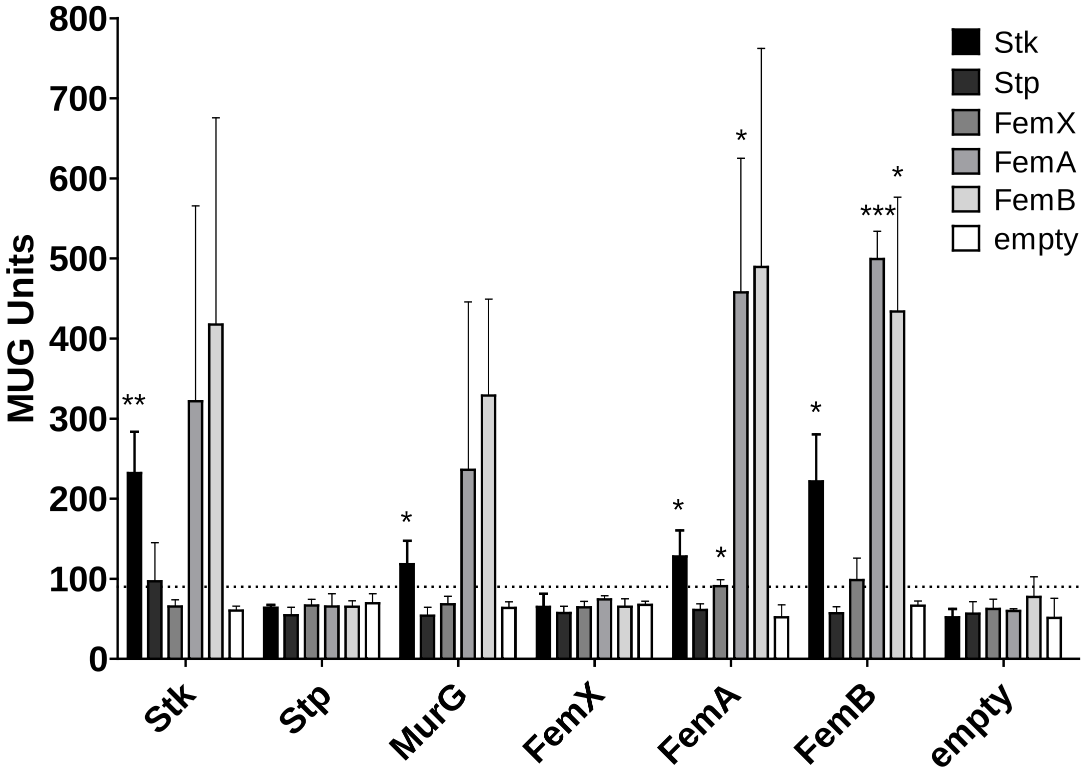

Figure S13. Quantitative measurement of selected protein interactions.

Measurement of β-galactosidase activity using fluorogenic substrate 4-methyllumbelliferyl β-D-galactopyranoside (MUG)[4] to quantify the protein interactions based on the spot assay (figure 4a). 6-10 transformants were grown in LB with antibiotics (100 µg/ml streptomycin and ampicillin + 50 µg/ml kanamycin) and 0.5 mM IPTG for 3 days at 18 °C. 20 µl culture was diluted in 80 µl Z-buffer and OD_{600} was measured. 25 µl 1 mg/ml MUG in DMSO was added. After 15 min incubation at room temperature 30 µl 1 M $NaC0_3$ was added and the fluorescence was measured (Ex. 355 nm, Em. 460 nm). Arbitrary units of β-gal activity (MUG units) were calculated as follows: $F_{355/460nm}$ / (t x OD_{600}). Significance values were calculated using a two-tailed unpaired student t-test (* $p < 0.05$, ** $p < 0.01$, *** $p < 0.001$). Horizontal dotted line indicate threshold of 90 MUG units. This threshold is based on the lowest value of a significant protein interaction (FemA-FemA).

Z-buffer: 8 g of $Na_2HPO_4x12H_2O$, 3.125 g of $NaH_2PO_4xH_2O$, 0.375 g KCl, 0.123 g $MgSO_4x7H_2O$ dissolved in 500 ml distilled Water)

Supplementary References

1 Mainiero, M. *et al.* Differential target gene activation by the *Staphylococcus aureus* two-component system saeRS. *Journal of bacteriology* **192**, 613-623, doi:10.1128/jb.01242-09 (2010).

2 Monk, I. R., Shah, I. M., Xu, M., Tan, M. W. & Foster, T. J. Transforming the untransformable: application of direct transformation to manipulate genetically *Staphylococcus aureus* and *Staphylococcus epidermidis*. *mBio* **3**, doi:10.1128/mBio.00277-11 (2012).

3 Steele, V. R., Bottomley, A. L., Garcia-Lara, J., Kasturiarachchi, J. & Foster, S. J. Multiple essential roles for EzrA in cell division of *Staphylococcus aureus*. *Molecular microbiology* **80**, 542-555, doi:10.1111/j.1365-2958.2011.07591.x (2011).

4 Vidal-Aroca, F. *et al.* One-step high-throughput assay for quantitative detection of beta-galactosidase activity in intact gram-negative bacteria, yeast, and mammalian cells. *BioTechniques* **40**, 433-434, 436, 438 passim (2006).
